# PanDrugs2: prioritizing cancer therapies using integrated individual multi-omics data

**DOI:** 10.1093/nar/gkad412

**Published:** 2023-05-19

**Authors:** María José Jiménez-Santos, Alba Nogueira-Rodríguez, Elena Piñeiro-Yáñez, Hugo López-Fernández, Santiago García-Martín, Paula Gómez-Plana, Miguel Reboiro-Jato, Gonzalo Gómez-López, Daniel Glez-Peña, Fátima Al-Shahrour

**Affiliations:** Bioinformatics Unit, Spanish National Cancer Research Centre (CNIO), Madrid 28029, Spain; CINBIO, Universidade de Vigo, Department of Computer Science, ESEI—Escuela Superior de Ingeniería Informática, 32004 Ourense, Spain; SING Research Group, Galicia Sur Health Research Institute (IIS Galicia Sur), SERGAS-UVIGO, 36213 Vigo, Spain; Bioinformatics Unit, Spanish National Cancer Research Centre (CNIO), Madrid 28029, Spain; CINBIO, Universidade de Vigo, Department of Computer Science, ESEI—Escuela Superior de Ingeniería Informática, 32004 Ourense, Spain; SING Research Group, Galicia Sur Health Research Institute (IIS Galicia Sur), SERGAS-UVIGO, 36213 Vigo, Spain; Bioinformatics Unit, Spanish National Cancer Research Centre (CNIO), Madrid 28029, Spain; Bioinformatics Unit, Spanish National Cancer Research Centre (CNIO), Madrid 28029, Spain; CINBIO, Universidade de Vigo, Department of Computer Science, ESEI—Escuela Superior de Ingeniería Informática, 32004 Ourense, Spain; SING Research Group, Galicia Sur Health Research Institute (IIS Galicia Sur), SERGAS-UVIGO, 36213 Vigo, Spain; Bioinformatics Unit, Spanish National Cancer Research Centre (CNIO), Madrid 28029, Spain; CINBIO, Universidade de Vigo, Department of Computer Science, ESEI—Escuela Superior de Ingeniería Informática, 32004 Ourense, Spain; SING Research Group, Galicia Sur Health Research Institute (IIS Galicia Sur), SERGAS-UVIGO, 36213 Vigo, Spain; Bioinformatics Unit, Spanish National Cancer Research Centre (CNIO), Madrid 28029, Spain

## Abstract

Genomics studies routinely confront researchers with long lists of tumor alterations detected in patients. Such lists are difficult to interpret since only a minority of the alterations are relevant biomarkers for diagnosis and for designing therapeutic strategies. PanDrugs is a methodology that facilitates the interpretation of tumor molecular alterations and guides the selection of personalized treatments. To do so, PanDrugs scores gene actionability and drug feasibility to provide a prioritized evidence-based list of drugs. Here, we introduce PanDrugs2, a major upgrade of PanDrugs that, in addition to somatic variant analysis, supports a new integrated multi-omics analysis which simultaneously combines somatic and germline variants, copy number variation and gene expression data. Moreover, PanDrugs2 now considers cancer genetic dependencies to extend tumor vulnerabilities providing therapeutic options for untargetable genes. Importantly, a novel intuitive report to support clinical decision-making is generated. PanDrugs database has been updated, integrating 23 primary sources that support >74K drug–gene associations obtained from 4642 genes and 14 659 unique compounds. The database has also been reimplemented to allow semi-automatic updates to facilitate maintenance and release of future versions. PanDrugs2 does not require login and is freely available at https://www.pandrugs.org/.

## Graphical Abstract

**Graphical Abstract:**
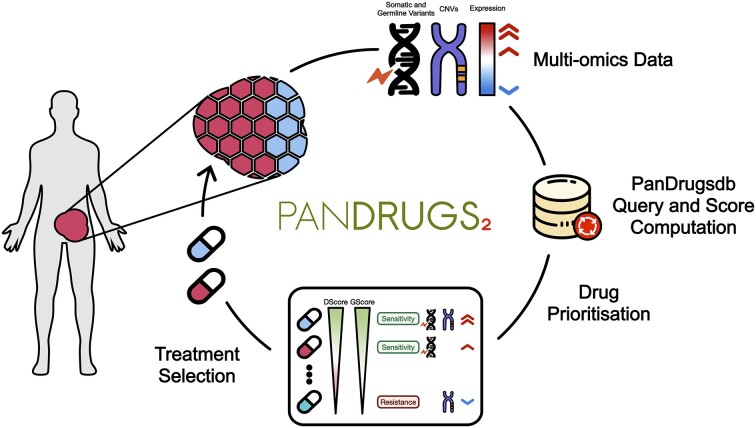

## INTRODUCTION

Identifying the most appropriate therapies from multi-omics data is a major challenge in cancer precision medicine. As our understanding of disease processes becomes more complex, it is increasingly clear that no single data type can provide a complete picture of disease pathogenesis or treatment response (1). This complexity leads to the detection of numerous molecular alterations within a single tumor including mutations, structural alterations and deregulated genes. As a result, researchers and clinicians face long lists of alterations which are hardly interpretable since most of them are clinically ‘unactionable’, irrelevant to tumor biology or their biological function is unknown, hindering the implementation of precision medicine strategies (2). In precision oncology, somatic mutations are commonly emphasized for clinical diagnosis and treatment. The joint evaluation of a patient's genomic and transcriptomic data can provide additional biological insights, including the determination of the functionality of a mutation (3,4). However, the application of multi-omics data to clinical practice remains daunting (5,6). In this scenario, *in silico* drug prescription tools have emerged to propose new data-driven therapeutic strategies based on the molecular features of the tumors, functional activity and drug sensitivity data (7). Some of these methodologies have focused on interpreting cancer genomic landscapes either by exclusively examining well-known clinically actionable variants (8–10) or by utilizing systems biology approaches to anticipate therapeutic response or uncover new druggable genes (11–13).

PanDrugs is a web tool to assist in selecting therapies based on the results of cancer genome-wide studies (14). It identifies actionable mutations and prioritizes drugs by calculating a Gene Score (GScore) and a Drug Score (DScore) that combine both biological and clinical relevance. PanDrugs represented the first drug prescription strategy applying a rationale based on pathway context, multi-gene markers impact and functional experiments. PanDrugs has been systematically applied to analyze cancer genomes in PCAWG (15), for predicting drug response in TCGA data (16) and for determining therapies in a T-ALL patient case report (17). The tool is included in the ELIXIR bio.tools catalogue and in the European Open Science Cloud (EOSC) marketplace.

Here, we present PanDrugs2, a major PanDrugs upgrade that is the result of five years of use and the feedback from our research and clinical users. PanDrugs2 has been specifically designed for precision medicine, which requires up-to-date user-friendly tools, seamless integration of multi-omics data for treatment prioritization and intuitive genomic reports to support clinical decision-making.

## OVERVIEW AND UPDATES OF CORE PANDRUGS FUNCTIONS

**Figure 1.**
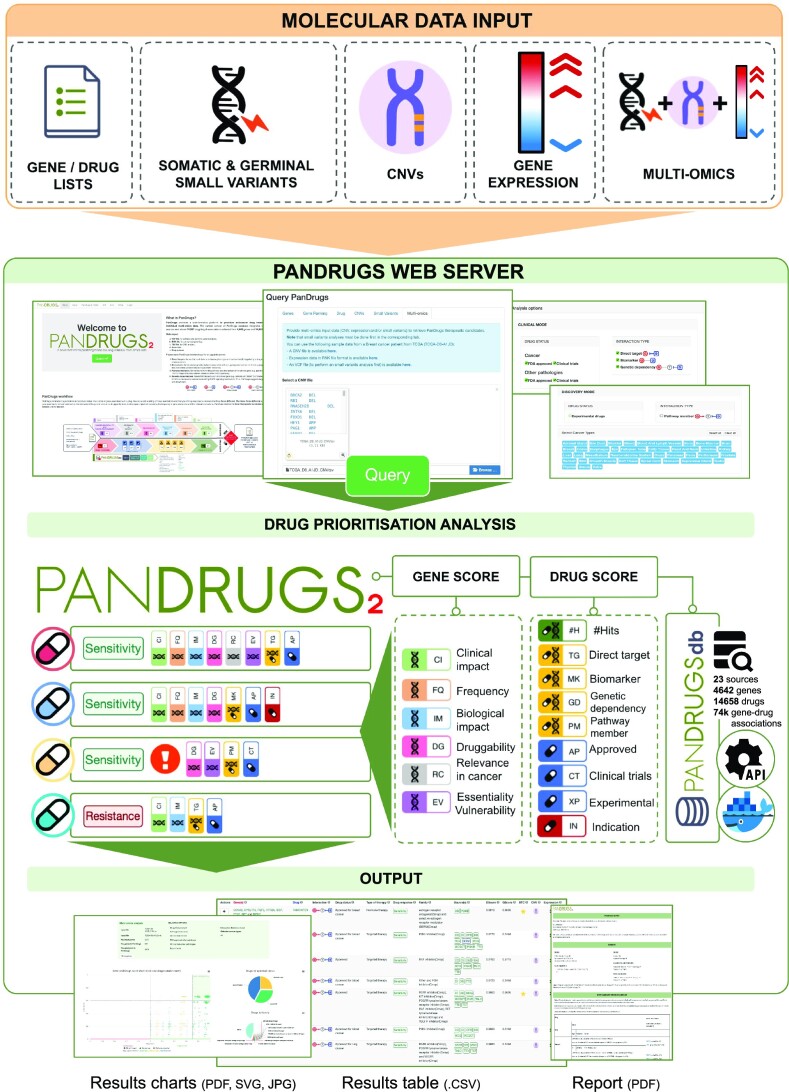
PanDrugs2 workflow. The PanDrugs2 web server allows uploading lists of genes, drugs, somatic and germline variants (.vcf), CNVs (.tsv) and gene expression data (.rnk) for drug prioritization analyses. Drug prioritization is performed using the GScore and DScore, which are calculated from the drug and gene annotations stored in PanDrugsdb. The results are presented as interactive tables and plots, as well as user-friendly reports to support clinical decision-making.

PanDrugs2 retains its core features while introducing new functionalities: (i) it now enables the integration of germline variant analysis for drug recommendations, (ii) accepts input data from copy number variants (CNVs) and/or transcriptomics experiments, (iii) supports multi-omics analyses combining somatic and germline single-nucleotide variants (SNVs), CNVs and gene expression data simultaneously, (iv) includes cancer genetic dependencies to support druggability evidence, and (v) generates user-friendly reports for clinical decision-making. PanDrugs database (PanDrugsdb) also includes new sources and has been redesigned to facilitate updating (Figure 1).

### Updates on PanDrugsdb

PanDrugsdb new version integrates drug-gene associations retrieved from 23 annotation sources supporting >74K associations obtained from 4642 genes and 14 659 unique compounds (Supplementary Figures S1 and S2; Supplementary Table S1). Importantly, the PanDrugsdb update process has been semi-automated including the steps of data downloading, harmonization and full integration of all gene and drug annotation sources. PanDrugsdb is manually curated to correct drug name inconsistencies and unify the Food and Drug Administration (FDA) and the European Medicines Agency (EMA) drug indications. The whole database can now be fully updated in less than a week, compared to several weeks in previous versions, facilitating ongoing maintenance and the release of future updates. PanDrugsdb has been upgraded to GRCh38-hg38 human genome assembly. PanDrugs API and the Docker image have also been updated accordingly.

### Updates on PanDrugs web-server

#### Updates in gene score and drug score

PanDrugs2 mines Pandrugsdb to calculate two scores for prioritizing treatments: The Gene Score (GScore) and the Drug Score (DScore). In PanDrugs2, new GScore and DScore are computed using the novel sources incorporated in PanDrugsdb (Supplementary Figure S3). The GScore evaluates for each gene alteration, the biological consequence and functional impact, the population allele frequency, the gene essentiality in cancer, the gene druggability and the clinical implication (Supplementary Tables S3 and S4). The GScore ranges from 0 to 1, with higher values corresponding to the most relevant and actionable targets. The DScore evaluates the suitability for each drug considering drug response (sensitivity or resistance), drug indication (for cancer or other diseases), clinical status (approved by the FDA or the EMA, in clinical trials or experimental treatments), type of gene-drug relationship, number of curated databases supporting this relationship and the collective gene impact (Supplementary Table S2). The range of the DScore is from –1 to 1, where negative values indicate resistance and positive values indicate drug sensitivity.

#### PanDrugs2 supports diverse omics input data types

PanDrugs2 new multi-omics analysis interface allows users to select the type of input data they want to combine (i.e. SNVs, CNVs and/or gene expression) and generate an integrated final results table (Supplementary Figures S4 and S5). Users can make queries using somatic SNVs to prioritize drug candidates whose direct (or indirect) gene targets have actionable alterations. Moreover, they can simultaneously incorporate pharmacogenetic information through germline analysis to detect variants which can be used to inform treatment decisions. CNV status and gene expression information (i.e. amplifications, deletions, over- and under-expression) are also incorporated in a new column of the drug prioritization results table. The table of results includes the candidate drugs for targeting the molecular alterations identified in all the inputs used.

#### PanDrugs2 evaluates pharmacogenetic variants for drug recommendations

PanDrugs now integrates the Pharmacogenomics Clinical Annotation Tool (PharmCAT) that extracts germline variants from an input VCF (Variant Calling File), interprets the variant alleles and generates a pharmacogenetics report with treatment recommendations (18) (Supplementary Figures S4 and S5). This enables the inclusion of guidelines from the Clinical Pharmacogenetics Implementation Consortium (CPIC) in a new column of the PanDrugs results featuring CPIC recommendations (e.g. ‘strongly not recommended’ drug). The full PharmCAT report can also be downloaded from the summary box at the top of the results page.

#### Incorporating genetic dependencies for drug prescription

PanDrugs2 classifies altered genes according to their druggability as ‘direct targets’, ’biomarkers’, ’genetic dependencies’ and ’pathway members’. The term ‘direct targets’ refers to genes that directly contribute to the disease phenotype and can be targeted by a drug, such as small molecules or monoclonal antibodies. ‘Biomarkers’ are genes with a genetic status associated with drug response, but the protein product itself is not the target of the drug. The term ‘pathway member’ encompasses all druggable downstream targets that are part of the underlying pathway of the user's input. In this new version, we have introduced ‘genetic dependency’ as a new druggability evidence obtained from CRISPR-Cas9 genome-wide loss-of-function screens in cancer cell lines (19). PanDrugs2 proposes drugs to target 361 genetic dependencies which would expand the therapeutic options with 171 approved drugs and 142 in clinical trials. Both ‘pathway member’ and ‘genetic dependency’ represent innovative approaches that open up new therapeutic opportunities for untargetable genes.

#### PanDrugs2 prioritizes therapies combining multi-omics data

PanDrugs2 can be executed using SNVs, CNVs and gene expression data simultaneously. The drug prioritization process combines the GScore and DScore to rank drugs targeting the queried altered genes (Supplementary Figure S5). Additionally, the server uses a ‘collective gene impact’ analysis to determine the number of druggable genes (direct targets, biomarkers, genetic dependencies and pathway members) associated with a particular drug. Thus, the drugs capable of targeting the greatest number of druggable genes are given priority. The final result is a list of drug-gene(s) associations ranked by DScore and GScore. An ideal multi-omics analysis would give the highest scores to those drug-gene associations consisting of approved drugs targeting direct actionable genes that are mutated, amplified and/or overexpressed. The final results table would highlight the Best Therapeutic Candidates or BTCs (GScore > 0.6 and DScore > 0.7) which would be included in the PanDrugs2 report.

#### PanDrugs2 genomic report

PanDrugs2 incorporates a new functionality to generate an automated, downloadable and easy-to-understand genomic report to assist in clinical decision-making. The report comprises two sections: (i) a brief summary and statistical overview of PanDrugs2 results and (ii) the complete list of BTCs annotated with their approval status, type of therapy, response, drug family, their actionable variants and the type of drug-gene association.

## IMPLEMENTATION

### PanDrugs2 web-server

Implemented in AngularJS 1.6, the PanDrugs2 front-end application is in charge of getting user queries, communicating with the back-end and displaying results in a user-friendly interface. The back-end application, implemented in Java with the JAX-RS API running in a Java EE application in Apache Tomcat 8, is in charge of (i) storing gene and drugs data, (ii) performing and managing multi-omics analyses and (iii) allowing interoperability with external applications through a public REST API.

### PanDrugsdb

The PanDrugs2 database is stored in MySQL 5.7 RDBMS. VCF variant analyses are performed with a Perl script, after an annotation step using the VEP (Ensembl Variant Effect Predictor, v109.3). If requested, germline variants are analyzed with PharmCAT (v2.1.2).

## RESULTS

PanDrugs2 GUI has been redesigned to support six types of queries in different file formats: gene list (.txt), gene ranking (.rnk), drug, CNVs (.tsv), small variants (.vcf) and multi-omics (.vcf/.tsv/.rnk). Additionally, the GUI enables two novel query modes: (i) a ‘clinical mode’ to prescribe drugs potentially applicable in the clinical setting (approved and in clinical trials) via direct target, biomarker and genetic dependency evidence and (ii) ‘discovery mode’ that also allows the prescription of experimental drugs and/or pathway members evidence. PanDrugs2 results table includes new icons showing mutational status, CPIC indications, CNV status and gene expression levels. Moreover, the web server displays updated results for the TCGA cohort using PanDrugs2.

### Use case 1: PanDrugs2 multi-omics analysis of a melanoma patient

**Figure 2.**
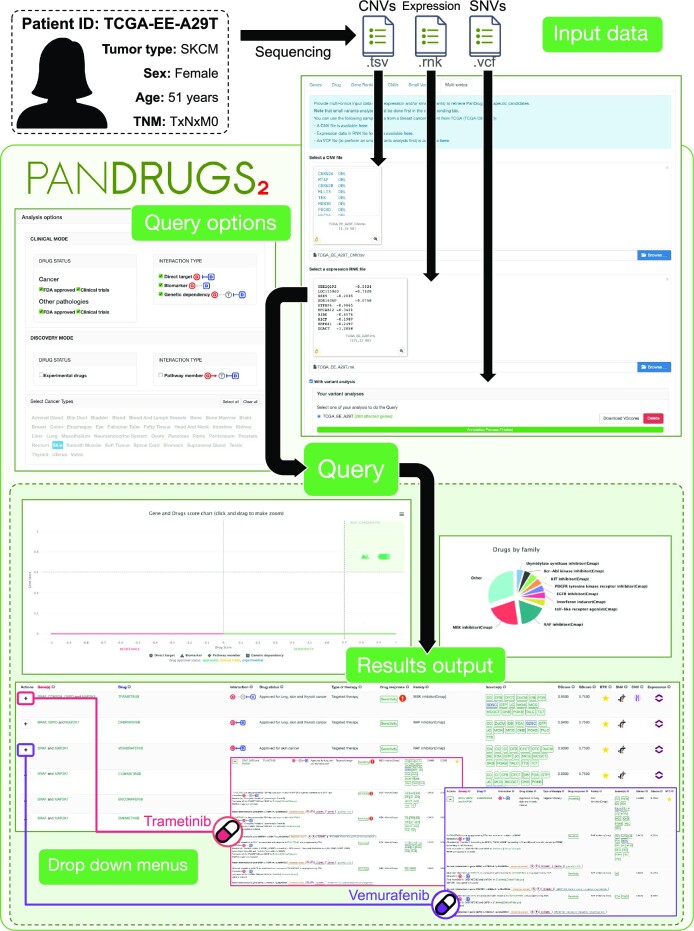
Use case 1: PanDrugs2 multi-omics analysis of a melanoma patient. Patient's CNVs (.tsv), gene expression (.rnk) and mutation (.vcf) data were analyzed simultaneously. The query was performed using the default parameters of the clinical mode and by selecting the patient's tumor type (i.e. skin). The results of PanDrugs2 analysis identified FDA-approved drugs, including MEK-targeted inhibitors (e.g. trametinib) and RAF-targeted inhibitors (e.g. vemurafenib), which are used to treat melanoma patients with *BRAF* mutations. MTOR inhibitors were also proposed when a drug-repurposing analysis was performed selecting all tumor types (data not shown).

A 51-year old female patient with metastatic melanoma from TCGA cohort (TCGA-EE-A29T) was found to have a somatic oncogenic *BRAF* mutation. The patient was treated with vemurafenib, which resulted in minimal toxicity and a complete response to therapy. We obtained from cBioPortal (20) patient′s multi-omics data (somatic SNVs, CNV, and expression data) and processed the file formats to ensure compatibility with PanDrugs2. We selected the ‘clinical mode’ and specified ‘Skin’ as the cancer type. PanDrugs2 results showed as BTCs: RAF inhibitors (e.g. vemurafenib) and MEK inhibitors (e.g. trametinib), which are FDA approved drugs, alone or in combination, for the treatment of *BRAF*-mutant melanoma patients. PanDrugs2 suggests vemurafenib as it directly targets the *BRAF* oncogenic mutation and because of the presence of mutated biomarkers associated with drug sensitivity (*MAP2K1* mutation and overexpression). Additionally, trametinib is also recommended by *BRAF* and *MAP2K1* alterations, as well as new molecular evidence, such as *G6PD* (mutation and high overexpression), *CDKN2A* (deletion and underexpression) and due to the genetic dependency of *MAP2K1* in *BRAF*-mutant cancer cell lines (Figure 2). Interestingly, when expanding the drug search to other types of cancer, PanDrugs2 suggests MTOR inhibitors like everolimus and sirolimus due to the presence of an *MTOR* oncogenic mutation. These findings suggest treating the patient with vemurafenib and the potential benefit of using MTOR inhibitors as a second-line treatment.

### Use case 2: PanDrugs2 small variants analysis of a breast cancer patient

**Figure 3.**
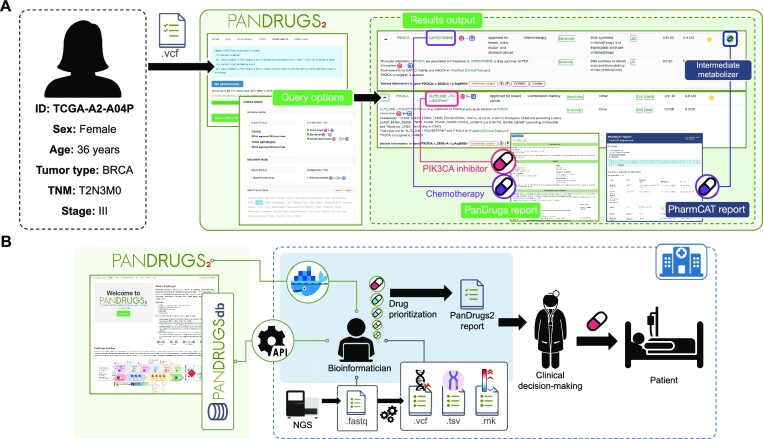
(**A**) Use case 2: Small variants analysis of a breast cancer patient. PanDrugs2 suggested a combination of alpelisib (PIK3CA inhibitor) + fulvestrant (estrogen receptor antagonist) based on the patient's somatic mutations. The recommended chemotherapies (i.e. capecitabine, gemcitabine, and docetaxel) were, in fact, the treatment regimens the patient had already received. PharmCAT identified fluorouracil and capecitabine as ‘Moderately recommended’ due to a heterozygous germline mutation in DPYD, which results in decreased drug metabolism. (**B**) PanDrugs2 integration in the clinical setting. PanDrugs2 can be easily employed in the clinical context either by performing automated queries to PanDrugsdb through its API or locally through its Docker image. The goal is to support clinical decision-making through a report that is easily interpretable by medical professionals.

We analyzed a TCGA patient (TCGA-A2-A04P) diagnosed with breast invasive ductal carcinoma with an oncogenic somatic mutation in *PIK3CA*. After diagnosis, the patient received radiotherapy and paclitaxel as adjuvant therapy followed by several cycles of chemotherapies used to treat breast cancer after recurrence. We applied PanDrugs2 using a combined analysis of the patient′s somatic and germline variants selecting the ‘clinical mode’ and ‘Breast’ cancer type. According to the results of PanDrugs2, alpelisib, a *PIK3CA* inhibitor, and its combination with fulvestrant are considered the most promising treatment options based on the patient's somatic mutations. Additional BTCs were chemotherapies approved for breast cancer such as capecitabine, gemcitabine and docetaxel, which were in fact the same treatment regimens administered to the patient. Interestingly, due to a heterozygous germline mutation in *DPYD* that leads to reduced drug metabolism, PharmCAT highlighted fluorouracil and capecitabine as ‘Moderately recommended’. Overall, PanDrugs2 results indicate that a personalized treatment approach incorporating a targeted therapy guided by *PIK3CA*-mutation and an adjusted dose recommendation to minimize toxicities based on a *DPYD* pharmacogenetic variant could have been beneficial for the patient (Figure 3A).

## DISCUSSION

*In silico* drug prescribing and multi-omics data integration can enhance personalized precision medicine by offering drugs tailored to individual molecular profiles, allowing a better patient stratification that entails more accurate diagnosis and treatments, and discovering new biomarkers for predicting drug response. Here, we have introduced PanDrugs2, a versatile *in silico* drug prescription tool which uniquely integrates and analyses multiple types of omics data, including small variants, CNVs, and transcriptomics. This innovative method expands the therapeutic possibilities by considering a wider spectrum of molecular alterations beyond the traditional one-size-fits-all approach (21). Based on feedback from PanDrugs’ users, we have implemented additional enhancements including pharmacogenetics analysis using germline variants that, together with somatic mutations, can yield substantially improved predictions of personalized drug efficacy. Furthermore, PanDrugs2’s new interface is tailored towards clinical and research purposes producing an intuitive multi-omics report that highlights the most promising drug candidates to support therapeutic decisions. Lastly, PanDrugsdb has undergone updates and reimplementation to enable semi-automated updates, simplifying future upgrades. At present, PanDrugs2 stands as the only *in silico* prescription approach that utilizes a logical framework based on multi-omics markers, genetic dependencies, molecular pathway context, and pharmacological evidence to systematically guide new tailored treatments. Two real-world use cases utilizing patient multi-omics data are presented to demonstrate PanDrugs2 usefulness. In the first use case, a melanoma patient, PanDrugs2 recommended the standard of care and second-line treatment options, highlighting the added value of the use of multi-omics data rather than just using mutations. In the second use case, we showed how a somatic mutation can guide personalized treatment of breast cancer and that the assessment of pharmacogenetic germline markers can help in choosing the treatment starting dose emphasizing the role of germline variants in precision medicine.

Despite its potential benefits, the application of *in silico* drug prescription in real clinical scenarios has not been accomplished yet. This is due to various reasons including the data analysis and interpretability bottlenecks and the requirement for comprehensive clinical data in large-scale multi-omics data sets to train and test the algorithms (22). The main objective behind the development of PanDrugs2 is to enhance data interpretability for clinical decision-making, thereby closing the gap between research and clinical applications. Furthermore, to improve interoperability, PanDrugs2 implements a Docker container and a REST-API to facilitate integration into standard sequencing data analysis pipelines commonly used in clinical settings (Figure 3B).

Although PanDrugs2 offers a valuable methodology, there is still a need for further efforts to advance cancer treatment by proposing more effective drugs and anticipating drug resistance. For instance, to improve drug prioritization and accuracy of prescription, additional biological relationship layers like protein interaction networks and transcriptional regulatory modules should be integrated, alongside a comprehensive drug ontology containing annotations such as drug indication, mechanism of action and side effects. Overall, integrating *in silico* drug prescribing with multi-omics data has immense potential to enhance precision medicine. While there are still challenges to be addressed, continued research and development in this area will likely lead to significant advancements in the years to come.

## DATA AVAILABILITY

PanDrugs2 is an open-source web server freely available at https://www.pandrugs.org/. No login is required for use. PanDrugsdb's code is available at https://github.com/cnio-bu/pandrugs-db (permanent DOI: 10.5281/zenodo.7892104).

## Supplementary Material

gkad412_Supplemental_File
