# Supplementary material for: PanDrugs2: prioritizing cancer therapies using integrated individual multi-omics data
Source: Nucleic Acids Res. 2023 May 19;51(W1):W411–8. doi: 10.1093/nar/gkad412 (PMC10320188; doi:10.1093/nar/gkad412)
Supplement: gkad412_Supplemental_File [file gkad412_supplemental_file.pdf]

SUPPLEMENTARY MATERIALS AND METHODS

PanDrugs2: prioritizing cancer therapies using integrated individual multi-omics data

María José Jiménez-Santos†, Alba Nogueira-Rodríguez†, Elena Piñeiro-Yáñez, Hugo López-Fernández, Santiago García-Martín, Paula Gómez-Plana, Miguel Reboiro-Jato, Gonzalo Gómez-López, Daniel Glez-Peña* and Fátima Al-Shahrour*

Table of contents

SUPPLEMENTARY TEXT **2**
MATERIAL AND METHODS 2
Drug-gene data sources 2
Drug name standardization 3
Annotations 3
Drug annotations 3
Gene annotations 4
Drug-gene annotations 6
Data integration 6
PanDrugs2 queries 7
DScore calculation 7
GScore calculation 8
PanDrugs2 prioritization process 10
RESULTS 11
Analysis of TCGA data 11
Use case 1: PanDrugs2 multi-omics analysis of a melanoma patient 11
Use case 2: PanDrugs2 small variants analysis of a breast cancer patient 11
SUPPLEMENTARY FIGURES **12**
Supplementary Figure S1 12
Supplementary Figure S2 13
Supplementary Figure S3 14
Supplementary Figure S4 15
Supplementary Figure S5 17
SUPPLEMENTARY TABLES **18**
Supplementary Table S1 18
Supplementary Table S2 20
Supplementary Table S3 21
Supplementary Table S4 22
SUPPLEMENTARY REFERENCES **23**

SUPPLEMENTARY TEXT

MATERIAL AND METHODS

Drug-gene data sources

PanDrugs database (PanDrugsdb) has been updated with drug-gene associations retrieved from 23 different sources (Supplementary Figure S1, Supplementary Table S1):

CIViC: Clinical Interpretation of Variants in Cancer (1) is an expert-crowdsourced knowledge base that, for some of the stored variants, contains information about treatment response. From this resource, using its Application Programming Interface (API) v2022-07-01, we retrieved drug-gene associations along with the specific alteration involved in the drug response. We only kept those entries with an accepted status and a validated evidence level.

DGIdb: The Drug Gene Interaction Database (DGIdb) (2) is a catalogue of standardized drug-gene interactions and druggability data mined from 41 well-known databases. We accessed DGIdb v4.2.0 programmatically through its API and retrieved information for all the human genes in GENCODE v39 (3). From this search, we kept the drug-gene associations from 17 sources: Cancer Commons (4), ChemblInteractions (5), Clearity Foundation Biomarkers (6), Clearity Foundation Clinical Trial (7), Database of Curated Mutations (DoCM) (8), Drug Target Commons (DTC) (9), FDA Pharmacogenomic Biomarkers (10), Guide to Pharmacology (11), The Jackson Laboratory Clinical Knowledgebase (JAX-CKB) (12), My Cancer Genome and My Cancer Genome Clinical Trial (13), NCI Cancer Gene Index (14), The Pharmacogenomics Knowledgebase (PharmGKB) (15), Targeted Agents in Lung Cancer (TALC) (16), The Druggable Genome Clinical Trial (TdgClinicalTrial) (17), Trends in the Exploitation of Novel Drug Targets (TEND) (18) and the Therapeutic Target Database (TTD) (19).

DrugBank: DrugBank (20) is a database that contains drug information including drug targets, commercial names and approval status. We downloaded DrugBank v5.1.9 as an XML (Extensible Markup Language) file and processed it using the R package dbparser v1.2.0 (21) to obtain a table with drug-gene associations.

GDSC: The Genomics of Drug Sensitivity in Cancer (GDSC) database (22) is a resource of drug sensitivity data in human cancer cell lines linked to genomics markers of drug response. We retrieved GDSC's ANOVA Results and Genetic Features tables for the Pan-Cancer set (release 8.2). We kept those drug-gene associations with a feature p-value < 0.001 and an FDR (False Discovery Rate) $<$ 0.25.

MOAlmanac: The Molecular Oncology Almanac (MOAlmanac) (23) includes a knowledge base of patient genomics data associated with drug response and prognostic information. We retrieved drug-gene associations with known sensitivity or resistance response from MOAlmanac database (2022 March 3 release). We kept validated records with some level of evidence, depending on the type of therapy:

- **For one-drug therapies:** We kept records with FDA-approved, guideline, clinical trial or clinical evidence.
- **For multi-drug therapies:** We kept records with FDA-approved or guideline evidence.

OncoKB: OncoKB (24) is an expert-curated precision oncology knowledge base that annotates the biological and oncogenic effects and the prognostic and predictive significance of somatic molecular alterations. We downloaded the actionable genes from its web page (v3.14) and selected those associations with top evidence (levels 1, 2 and R1), preserving the information about the specific alterations involved in the drug response.

Thera-SAbDab: The Therapeutic Structural Antibody Database (Thera-SAbDab) (25) contains antibody and nanobody therapy information, including their targets and approval status. We downloaded the full database on the 21st of June 2022 and manually formatted some target genes to HGNC (HUGO Gene Nomenclature Committee) v2022-10-01 (26) symbols before constructing PanDrugsdb.

Drug name standardization

The same compound can be referred to by different names in distinct databases. Thus, in order to integrate annotations from the aforementioned sources into PanDrugdb, drug names had to be standardized. For each source drug name, PanDrugs2 retrieves a *standard_drug_name*, which is the primary key of PanDrugsdb, and a *show_drug_name*, which is the term that is shown in PanDrugs2 ranking. Each unique *standard_drug_name* has a corresponding unique *show_drug_name* and vice versa.

PanDrugsdb's compound standardization process consists of an automatic step and a manual review:

For the **automatic standardization process**, we used PubChemPy v1.0.4 (27) to retrieve all source drug names synonyms from PubChem (28) (accessed on the 29th of November 2022). Then, for each source drug name, we prioritized the International Nonproprietary Names (INN) as *show_drug_names*. If no INN was retrieved for a particular compound, its *show_drug_name* was set to the first synonym returned by PubChemPy. Likewise, in either case, the *standard_drug_name* was set to the first synonym returned by PubChemPy. If no synonym was found, both the *show* and *standard_drug_names* were set to the source drug name.

In the **manual review step,** we corrected inconsistencies that may have arisen during the automatic standardization process. Moreover, we assigned a curated *standard* and *show_drug_name* to those source drug names for which PubChemPy did not find any synonyms.

Annotations

Drug annotations

Drug status: We used Drugs@FDA (29) (accessed on the 14th of March 2022) and EMA (30) data (accessed on the 31st of May 2022), as well as information from ClinicalTrials.gov database (31) (accessed on the 15th of March 2022). These data were manually curated to assign a drug status to each compound in PanDrugsdb. This field was stratified into 5 main categories, 2 of them with subcategories (Supplementary Figure S2A):

- **Approved:** Drugs approved by the FDA and/or the EMA.
    - In cancer: Compounds indicated for cancer treatment.
    - Other and in cancer clinical trials: Drugs approved for other conditions or pathologies that are in cancer clinical trials.
    - Other: Drugs prescribed for other conditions or pathologies.
- **Clinical trials:** Drugs in clinical trials according to ClinicalTrials.gov database.
    - In cancer: Compounds that are under study in cancer clinical trials.
    - Other: Compounds in clinical trials for other conditions or pathologies.
- **Experimental:** Drugs in the pre-clinical stage.
- **Withdrawn:** Discontinued drugs according to the FDA and/or EMA.
- **Undefined:** When the drug name refers to a broad group of compounds (e.g. radiation therapy).

Type of therapy: Drugs approved for cancer treatment were manually classified into 7 different groups (Supplementary Figure S2B-C):

- **Chemotherapy:** Drugs that kill fast-growing cells.
- **Targeted therapy:** Drugs that specifically attack cancer cells. All compounds with names ending in "-ib" or "-mab" were automatically assigned to this category.
- **Hormone therapy:** Suppression of certain hormones that can prompt or help in tumour growth.
- **Immunotherapy:** Drugs that boost or change how the immune system works to fight against cancer.
- **Photodynamic therapy:** Use of light-sensitive drugs, called photosensitizing agents, along with light to kill cancer cells.
- **Combination therapy:** Use of drug combinations to treat cancer. These therapies are treated as one-drug therapies in PanDrugsdb creation, Drug Score (DScore) calculation and Gene Score (GScore) collapsing.
- **Other:** Drugs that do not belong to any of the previous groups.

Tumour indication for which each drug is prescribed. Drugs approved for cancer treatment were manually annotated based on FDA drug labels (32) (accessed on the 1st of June 2022) and EMA indications (Supplementary Figure S2D). Drugs in cancer clinical trials were assigned the label "cancer".

Drug family: In order to classify PanDrugsdb compounds into drug families, we retrieved data from KEGG BRITE's (33) hierarchical Target-based Classification of Drugs (br08310; v101) and the CLUE Repurposing app (v1.2; build 1.44) from the Connectivity Map (CMap) (34). The Target-based Classification of Drugs divides drugs into 10 general levels which are further subdivided into more specific groups. We defined the second most general level of this classification, together with the CMap's Mechanisms of Action (MoAs), as the drug family of each PanDrugsdb compound.

Gene annotations

Driver role: In order to retrieve the driver role of each gene in PanDrugsdb, we downloaded COSMIC's Cancer Gene Census (CGC) v95 (35) and OncoVar v1.2 (36) data for all cancer types in the TCGA project (37). OncoVar is a resource that integrates data from several driver gene sources, including but not limited to OncoKB, 2020Rule (38), CTAT (39), ONGene (40) and TSGene (41). We retrieved COSMIC (*Role in Cancer* column) and OncoVar data (*OncoKB*, *2020Rule*, *CTAT*, *Oncogene* and *TSgene* columns) to create a table with gene roles. "Fusion" labels coming from COSMIC were transformed into "Oncogene" annotations.

Next, we processed this gene role table to obtain PanDrugsdb driver role annotations. "Pssible_Oncogene" and "Pssible_TSG" annotations from CTAT were removed and genes were assigned one of these 3 driver role labels:

- **Oncogene:** If all sources with available data reported the gene as an oncogene.
- **Tumour suppressor gene:** If all sources with available data reported the gene as a tumour suppressor gene.
- **Unclassified:** If there was no agreement between different sources or the gene was not reported in any of them.

Pathways: We downloaded the collection of KEGG PATHWAY (33) maps for *Homo sapiens* (v103) to annotate the pathways in which each PanDrugsdb gene was involved.

Pathway members: We also downloaded the KEGG Markup Language (KGML) files for a list of *Homo sapiens* pathways to extract the upstream genes of each gene with a drug association in PanDrugsdb. We included the pathways listed in Hipathia (42).

We only conserved upstream genes that were within a maximum distance of 4 from the affected gene. To restrict the manifold linkages available, we checked the upstream gene in the gene role table and the linkage type with the affected gene, keeping only those entries that met the following criteria:

- The upstream gene had an unknown role or was reported by all sources with available data as an oncogene or a fusion. Moreover, this gene activated or induced the expression of the affected gene.
- All sources with available data reported the upstream gene as a tumour suppressor and this gene inhibited or repressed the expression of the affected gene.
- Entries with any other linkage type that has not been cited previously.
- Entries in which the upstream gene was dually reported as an oncogene and tumour suppressor or as a possible oncogene or possible tumour suppressor.

Genetic dependencies: Our rationale for identifying synthetic lethal alterations closely follows that of Perales-Patón J. and colleagues (43). Briefly, we integrated somatic single nucleotide variants (SNVs), copy number variants (CNVs), gene expression profiles and essentiality scores from cancer cell lines profiled in the Cancer Dependency Map (DepMap, release 22Q2) (44).

First, we performed a molecular stratification of cancer cell lines according to SNVs, CNVs and gene expression profiles. DNA alterations were mapped to GRCh38 coordinates using CrossMap (v0.6.3) (45) and classified according to their functional impact into activating (Gain of Function, GoF) or deleterious (Loss of Function, LoF):

- **LoF labels** were assigned to genes affected by homozygous variants (Variant Allele Frequency or VAF >= 0.7) whose consequence truncated the protein product (“Nonsense_Mutation”, “Frame_Shift_Ins”, “Missense_Mutation”, “Frame_Shift_Del”, “De_novo_Start_OutOfFrame”, "Nonstop_Mutation”, “Start_Codon_Del”, “Stop_Codon_Ins”, “Start_Codon_Ins”, “Stop_Codon_Del” annotations) or by a deletion (Copy Number ratio, CN ratio <= 0.5).
- **GoF labels** were assigned to genes amplified at a very high magnitude (CN ratio >= 8) and unaffected by any truncating mutation.

The particular case of somatic missense variants was handled by taking into account both the zygosity of the variant and the role of the affected gene in cancer as defined by the CGC:

- Heterozygous (VAF >= 0.2) missense variants affecting oncogenes were classified as GoF.
- Homozygous (VAF >= 0.7) missense variants affecting tumour suppressor genes were classified as LoF.

Functional LoF/GoF events spanning solely from CNVs were further refined by interrogating their impact on the expression profile of the affected cell lines. To do so, we grouped cancer cell lines harbouring CNVs of the same gene with the same functional event (i.e. *MYC* GoF) and compared the normalised expression of the affected gene against wild-type cancer cell lines. Functional events in cell lines where the affected gene was overexpressed (z-score > 1.96) or underexpressed (z-score <= -1.96) compared to the rest of the collection were kept for subsequent analysis.

Next, cancer cell lines sharing a common functional event were grouped for statistical comparison regardless of tissue of origin. To mitigate the impact of multiple passenger events co-occurring with a driver functional event, we further identified and filtered them based on the following criteria:

- Any GoF/LoF alteration present in a subset of a larger set of cancer cell lines that share an oncogenic dependency, thus being self-contained in a driver event and featuring an overlap of at least 80% of the cancer cell lines.

- Passenger concurrent alterations by CNVs of large segments of DNA spanning a driver event. These were defined as events occurring in the same cancer cell lines with the same type of functional impact (GoF or LoF, Jaccard index > 0.65) and located within 2Mb of the driver event.

To correlate LoF/GoF alterations in a gene A with increased dependency on the function of a gene B, we performed a gene set enrichment analysis with fgsea v1.24.0 (46). To do so, we first downloaded DepMap's 22Q2 Chronos (47) Gene Effect dataset, in which negative values indicate cell death or growth inhibition as a consequence of the gene knockout and thus higher essentiality. Then, we ranked cancer cell lines harbouring the same functional event by their essentiality score for each gene B, to test whether they were more likely to appear at the top of this ranking. To limit the number of genes B considered and reduce the number of tests to perform, we chose genes whose dependency scores showed a pan-cancer tendency towards essentiality (skewness <= 0.5), thus disregarding those with little to no evidence of being essential. Furthermore, we only considered cancer cell lines without an LoF of gene B. All tests were performed with 10,000 permutations, at least 5 cancer cell lines and one tail: the top of the rank. Gene A-B associations were considered significant if the adjusted p-value after FDR correction was <= 0.25.

Drug-gene annotations

Molecular alteration: This annotation contains information about the gene alterations that confer sensitivity or resistance to a given drug. These data were mined from CIViC, GDSC, MOAlmanac and OncoKB, as stated previously (Supplementary Table S1). Moreover, some alterations were manually annotated based on existing literature, such as *MET* amplifications conferring resistance to EGFR inhibitors. PanDrugsdb's molecular alterations include somatic and germline variants, point mutations, indels, gene fusions, CNVs, splice mutations, amplifications, duplications, deletions, rearrangements and promoter methylations.

Definition of direct drug target or biomarker: PanDrugsdb genes associated with the response to a drug were assigned one of these two labels:

- **Direct target:** Genes that contribute to a disease phenotype and can be directly targeted by the drug (e.g. *BRAF* is a direct target for vemurafenib).
- **Biomarker:** Genes whose genetic status is associated with a drug response according to clinical or pre-clinical evidence but are not the drug target themselves (e.g. *BRCA*-mutated cancers responding to PARP inhibitors).

Based on the type of information stored in each source database (Supplementary Table S1), we classified drug-gene associations from Cancer Commons, Clearity Foundation Clinical Trial, My Cancer Genome, TALC, TEND, TTD, Thera-SAbDab and DrugBank as targets. The rest of the associations were labelled as markers.

Drug response: By default, all drug-gene associations were assigned a sensitivity response. These annotations were refined using CIViC, GDSC, MOAlmanac and OncoKB, which include information about gene variants that confer resistance to specific drugs (Supplementary Table S1).

Data integration

In this second version of PanDrugs, we have tried to automate most of the database creation process. The code is available on our GitHub repository: https://github.com/cnio-bu/pandrugs-db. Briefly, we downloaded all sources, processed them and created two files for manual curation. After checking them, we constructed a candidate table that underwent further manual review in the specified entries. We also created a file with manually curated controlled records. After this second review, we made corrections using the controlled records and obtained the final PanDrugsdb table. This database contains 74,087

drug-gene associations obtained from 4,642 genes and 14,659 unique compounds (Supplementary Figure S3).

PanDrugs2 queries

There are five types of queries to obtain a rank of therapies according to an individualized genomics profile, depending on the input data:

- **Gene names query:** Using a list of altered genes as input in .txt format.
- **Gene ranking query:** Using a ranked list of altered genes in .rnk (Ranked list) format.
- **CNVs query:** Using a list of altered genes and their CNV status in .tsv (Tab Separated Values) format.
- **Small variants query:** Using a Variant Calling File (VCF) containing a set of somatic (and optionally germline) variants corresponding to the GRCh38 assembly of the human genome.
- **Multi-omics query:** Using at least two of these inputs:
    - A .tsv compatible with a CNVs query.
    - A VCF compatible with a small variants query.
    - A .rnk file with genes ranked according to their expression change between tumour and normal samples.

Another possibility is to make a **drug query** to find out which genes are associated with a particular drug.

Except for the drug queries, PanDrugs2 results can be filtered by drug status, the type of drug-gene association and the tumour indication.

- **By drug status:** Allows to filter therapeutic options according to their approval status (Approved, Clinical Trials or Experimental) for cancer or other pathologies.
- **By type of interaction:** Allows to filter therapeutic options according to whether the drug-gene association is direct or indirect.
    - Direct: The input genes are the direct targets or biomarkers of the drug.
    - Indirect: The input genes are a genetic dependency or an upstream gene of a direct target.
- **By tumour indication:** Allows to filter therapeutic options approved for specific tumour types.

DScore calculation

PanDrugs2 ranking is ordered by decreasing Drug Score (DScore), which measures the suitability of the treatment for a particular patient. It ranges from -1 to 1, with the negative values corresponding to resistance and the positive values corresponding to sensitivity.

PanDrugsdb stores a pre-computed DScore ($preDScore$) for each drug-gene association (Supplementary Figure S3). This score has been calculated according to the drug indication for cancer or other diseases, its approval status and the type of direct drug-gene association (direct target or biomarker). Moreover, the sign of the pre-computed DScore indicates the direction of the drug response (sensitivity or resistance) (Supplementary Table S2). Please note that combination therapies are treated as one-drug therapies.

The final DScore reported by PanDrugs2 depends on the type of query. Let A_d be the set of drug-gene associations a between a particular drug d and all queried genes $G = \{g_1, g_2, \dots, g_N\}$, such as $A_d = \{a | g \; associated \; with \; d\}$. Moreover, let D_d be the pre-computed DScores of elements in a, such as $D_d = \{preDScore_a | a \in A_d\}$. After a drug query, the ranking shows the pre-computed DScore of each drug-gene association a. After any other query, PanDrugs2 adds a variable x_a to each

$|preDScore_a|$. Then it identifies the element i with the maximum value such as $i = argmax(|preDScore_a| + x_a)$. Finally, it multiplies this value by the sign of $preDScore_i$ (Equation 1).

$$DScore_d = \begin{cases} preDScore_a & Drug\ Query \\ (|preDScore_i| + x_i) \cdot sgn(preDScore_i) & Otherwise \end{cases} \quad (1)$$

The value of the variable x_a depends on the drug status and the type of association with the gene (Equation 2):

- **For approved drugs or drugs in clinical trials:** x_a is computed based on the collective gene impact (CGI_d) and the database factor (DF_a). The collective gene impact is calculated as the number of drug-associated genes up to a maximum of 9, penalized if there are no direct associations (direct targets or biomarkers). The database factor is the number of curated sources C_a that report each association, up to a maximum of 9 (Equations 3-4).
- **For experimental drugs:** PanDrugs2 penalizes the DScore if all genes have indirect associations (genetic dependencies or pathway members) with the drug.

$$x_a = \begin{cases} -0.1 + 0.01 \cdot CGI_d + 0.001 + 0.001 \cdot DF_a & Not\ Experimental \\ -0.0002 & Experimental\ w/\ only\ indirect\ associations \\ 0 & Experimental\ w/\ any\ direct\ association \end{cases} \quad (2)$$

$$CGI_d = \begin{cases} min(|A_d|, 9) - 1 & If\ only\ indirect\ associations \\ min(|A_d|, 9) & Otherwise \end{cases} \quad (3)$$

$$DF_a = min(|C_a|, 9) \quad (4)$$

If there are two $preDScore_i$ with an opposed sign, the positive sign is chosen: $sgn(preDScore_i) = 1$. Moreover, if the mutation of a drug-associated gene confers resistance to the drug and the alteration of another gene indicates sensitivity, that drug will be assigned the label "Both".

GScore calculation

PanDrugs2 ranking is ordered by decreasing Gene Score (GScore), which measures the biological relevance of a gene in the tumoral process and its druggability. It ranges from 0 to 1, with higher values corresponding to more relevant and actionable targets.

PanDrugsdb stores a pre-computed GScore for each gene symbol (Supplementary Figure S3). This score has been calculated according to gene essentiality, tumour vulnerability, the relevance of the gene in cancer and its druggability level (Supplementary Table S3). To pre-compute PanDrugsdb GScores, we first downloaded data from OncoVar, DepMap and other two publications (48, 49):

OncoVar: We downloaded OncoVar data for all PanCancer genes in the TCGA project. In this table, each gene is assigned a Consensus Score and a Driver Level. The Consensus Score is computed as the number of different OncoVar sources that report the gene as a cancer driver, weighted by the relevance of each database. Based on this Consensus Score, the genes are assigned a Driver Level, which indicates the grade of pathogenicity of the gene (0: non-pathogenic, score = 0; 1: possible pathogenic, score = 1; 2: likely pathogenic, 1 < score <= 10 ; 3: probable pathogenic, 10 < score < 20; 4: pathogenic, score >= 20). For genes with a Driver Level <= 3, we scaled the Consensus Score between 0 and 0.5. Likewise, we scaled the Consensus Score between 0.5 and 1 for genes with a Driver Level = 4.

DepMap: We scaled each gene effect skewness across all tested cell lines between -0.5 and -2 from 0 to 1. Skewness values < -2 were considered saturated and assigned a scaled score of 1. Values > -0.5 were assigned to 0.

Cancer Hallmarks: We downloaded Supplementary Table S8 from Iorio F *et al.*, which contains the genes associated with each cancer hallmark. For each gene, we counted the number of different cancer hallmarks in which it was involved. Then, we multiplied this number by 0.2, up to a maximum value of 1. Genes involved in more than 5 cancer hallmarks were assigned the maximum value.

Target Development Levels (TDLs): We downloaded Supplementary Table S1 from Jiang J *et al.*, which contains each gene's TDL. This classification measures the druggability of each gene and is subdivided into 4 levels (Tclin: targets of approved drugs; Tchem: targets of small molecules; Tbio: genes that are not targets of drugs or small molecules; Tdark: genes with unknown biological function). We assigned the following scores to each group: Tclin = 1; Tchem = 0.5; Tbio = 0.25 and Tdark = 0.125.

Then, we calculated a pre-computed GScore ($preGScore$) for each gene g contained in $G = \{g_1, g_2, ..., g_N\}$ (Equation 5):

$$preGScore_g = 0.55 \cdot OncoVar_g + 0.15 \cdot DepMap_g + 0.1 \cdot Hallmarks_g + 0.2 \cdot TDLs_g \quad (5)$$

The final GScore reported by PanDrugs2 depends on the type of query (Equation 6):

- **Drug, gene names or CNVs query:** The GScore is equal to the pre-computed GScore.
- **Gene ranking query:** Let R be the set of ranking values such as $R = \{r_g | g \in G\}$. The GScore of each gene is computed by min-max scaling its ranking value between 0 and 1.
- **Small variants query:** The somatic variants included in the VCF are annotated using Ensembl's Variant Effect Predictor (VEP) v109.3 (50) and additional databases such as supplementary material from (51), ClinVar v2022-05 (52), COSMIC v96 (53), InterPro v88.0 (54), KEGG Pathways v103, Pfam v35.0 (55) and UniProt release 2022_01 (56). Each input gene is assigned a driver role according to the table obtained from COSMIC's CGC and OncoVar (see Gene annotations subsection for further information):
  - **Oncogene:** If all sources with available data report the gene as an oncogene.
  - **Tumour suppressor gene:** If all sources with available data report the gene as a tumour suppressor gene.
  - **Unclassified:** If the gene role is neither oncogene nor tumour suppressor, there is no agreement between different sources or the gene is not reported in any of them. If a variant has a consequence of "stop_gain", "stop_lost", "frameshift_variant", "splice_donor_variant", "splice_acceptor_variant" or "splice_region_variant", its corresponding gene is relabelled from unclassified to tumour suppressor gene.

  With these annotations, a Variant Score ($VScore$) is computed for each variant v taking into account its biological impact, its frequency, its clinical implications and the pre-computed GScore (Supplementary Table S4). The final GScore is then calculated as the maximum VScore among all variants affecting the gene principal isoform ($V_g = \{v_1, v_2, ..., v_N\}$), which is selected according to APPRIS database (57). If APPRIS does not report any principal isoform, V_g contains variants affecting all isoforms.

$$GScore_g = \begin{cases} preGScore_g & Drug,\ gene\ names\ or\ CNVs\ query \\ (r_g - min(R))/(max(R) - min(R)) & Gene\ ranking\ query \\ max\,\{VScore_v | v \in V_g\} & Small\ variants\ query \end{cases} \quad (6)$$

- **Multi-omics query:** There can be two types of GScores:
    - **For the variants in the VCF:** The GScore is computed from VScores as in a small variants query.
    - **For the genes with CNVs:** The GScore is computed as in a CNVs query.

  If a gene has small variants and CNVs, the GScore computed from VScores has priority over the other GScore.

Except for the drug queries, the final PanDrugs2 ranking shows a collapsed GScore ($colGScore$) for each treatment d (either one-drug or combined therapy), which is the maximum GScore among all the drug-associated genes in A_d (Equation 7). PanDrugs2 collapsed table can be expanded to retrieve each individual GScore.

$$colGScore_d = max\{GScore_g | g \in A_d\} \quad (7)$$

PanDrugs2 prioritization process

Based on the genetic alterations of a patient, PanDrugs2 queries PanDrugsdb and returns a prioritized table of drugs ranked by decreasing DScore and GScore. This table includes general annotations such as the input genes associated with the drug, type of drug-gene association and source, drug status, type of therapy, drug response and drug family. It can also contain annotations specific to the query type, such as the pharmacogenetics recommendation and SNV presence (multi-omics queries), CNV presence (CNVs and multi-omics queries) and expression level (multi-omics query) of the corresponding gene. PanDrugs2 resolves the Best Therapeutic Candidates (BTCs) as the drugs with a DScore > 0.7 and a GScore > 0.6 and labels them with a star icon in the final table. As the entries in this table show information collapsed by drug, the fields containing gene information can be expanded through a dropdown button.

Depending on the type of query, PanDrugs2 performs different steps before calling PanDrugsdb (Supplementary Figures S4-5):

- **In a gene names, gene ranking, CNVs or drug query,** the input list of genes or the input drug is directly used to query PanDrugsdb.
- **In a small variants query,** the somatic variants included in the VCF are annotated using VEP and additional databases and a VScore is computed as described in the GScore calculation section. An intermediate table containing all annotations is generated and only the genes affected by variants with a moderate or high impact according to VEP are used to query PanDrugsdb.

  In this type of query, it is also possible to call PharmCAT v2.1.2 (58) to include the Clinical Pharmacogenetics Implementation Consortium (CPIC) (59) guidelines in the final ranking returned by PanDrugs2. In order to do so, the input VCF must contain germline variants and genotype data. Optionally, the user can also upload a .tsv file with genotypes called outside PharmCAT. PanDrugs2 runs PharmCAT and parses its output to return a column with the pharmacogenetics recommendation for each drug. This categorization consists of 5 levels: "Strongly Recommended", "Moderately Recommended", "Warning", "Moderately not Recommended" and "Strongly not Recommended".

- **Multi-omics query:**

  If expression data is available, PanDrugs2 will compute the 90th percentile of the expression metric and will assign an expression label to each gene in the .rnk file:

    - **Highly Overexpressed:** Genes with an expression metric above the 90th percentile.

- **Overexpressed:** Genes with an expression metric > 0.
- **Underexpressed:** Genes with an expression metric < 0.

Then, PanDrugs2 will query its database with the genes with known CNV status information, if available. Moreover, if there is a VCF, PanDrugs2 runs a small variants query (with an optional call to PharmCAT) as detailed before. The output table will contain annotations for each drug-associated gene, including its SNV, CNV and expression status, when available.

RESULTS

Analysis of TCGA data

We retrieved the somatic alterations in MAF (Mutation Annotation Format) from the GDC data portal (60) for the 17 cancer types analyzed in the previous version of PanDrugs (61). To assign the tumour type to each case, we used the files with clinical information about patients aggregated by tumour type provided by the GDC Data Portal. These files also allowed us to know the total number of patients per type of tumour. We then converted the MAF files into the default VEP input format and used them to obtain VCF files via VEP v106. These VCF files were used as input to query PanDrugs2 and retrieve the results via API. Then, all files were joined together by tumour type and we kept only those records with a GScore > 0.6 and without evidence of resistance. With these records, we created tabulated files with all the metrics and information needed for drawing the bubble plots of the web page.

Use case 1: PanDrugs2 multi-omics analysis of a melanoma patient

From the GDC data portal, we retrieved the somatic alterations of TCGA-EE-A29T patient in a MAF file which was subsequently transformed into a VCF. The CNV data was retrieved from the patient's summary page in cBioPortal (62) (Skin Cutaneous Melanoma TCGA PanCancer Atlas). To obtain a .tsv compatible with PanDrugs2, we conserved "AMP" gene annotations and transformed "DeepDel" labels to "DEL". Expression data was also downloaded from the cBioPortal. We retrieved the file *data_mrna_seq_v2_rsem_zscores_ref_diploid_samples.txt*, which contains the relative expression (z-score) of each patient's gene to the gene's expression distribution in all diploid tumours for the gene in question in Skin Cutaneous Melanoma TCGA PanCancer data. We kept the z-scores of all HGNC symbols with available expression for patient TCGA-EE-A29T and collapsed duplicated values to the maximum.

Use case 2: PanDrugs2 small variants analysis of a breast cancer patient

We retrieved a VCF with the patient's germline variants and genotypes from (63). Moreover, from the GDC data portal, we retrieved the somatic alterations of TCGA-A2-A04P patient in a MAF file which was subsequently transformed into a VCF.

SUPPLEMENTARY FIGURES

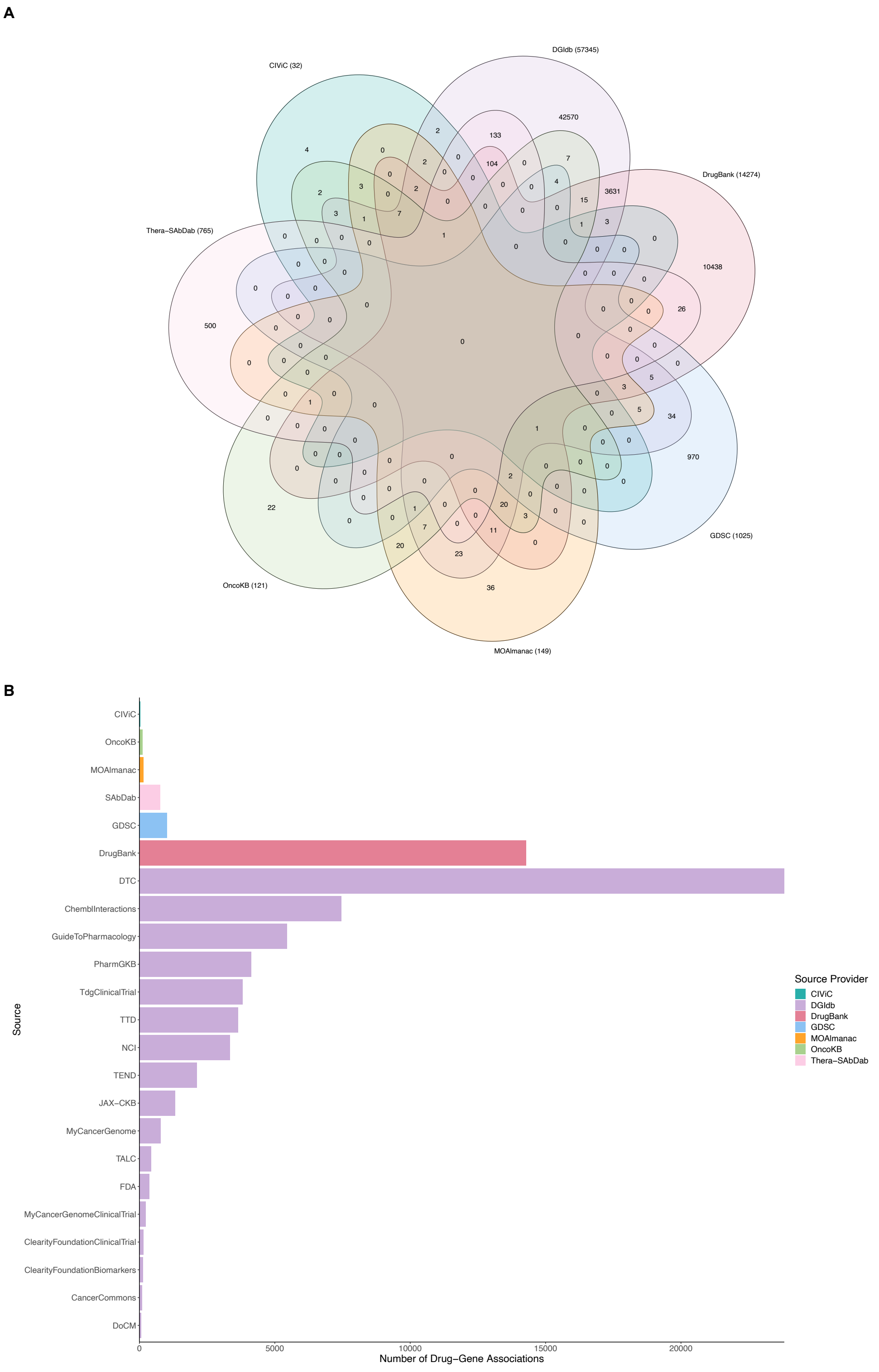

Supplementary Figure S1. PanDrugsdb drug-gene association sources. (A) **Overlapping between the 7 source providers.** The total number of associations retrieved from each source provider is shown in parenthesis next to its name. (B) **Number of drug-gene associations retrieved from each source.** The 17 sources included in DGIdb are shown separately. ***PanDrugsdb:*** *PanDrugs database.*

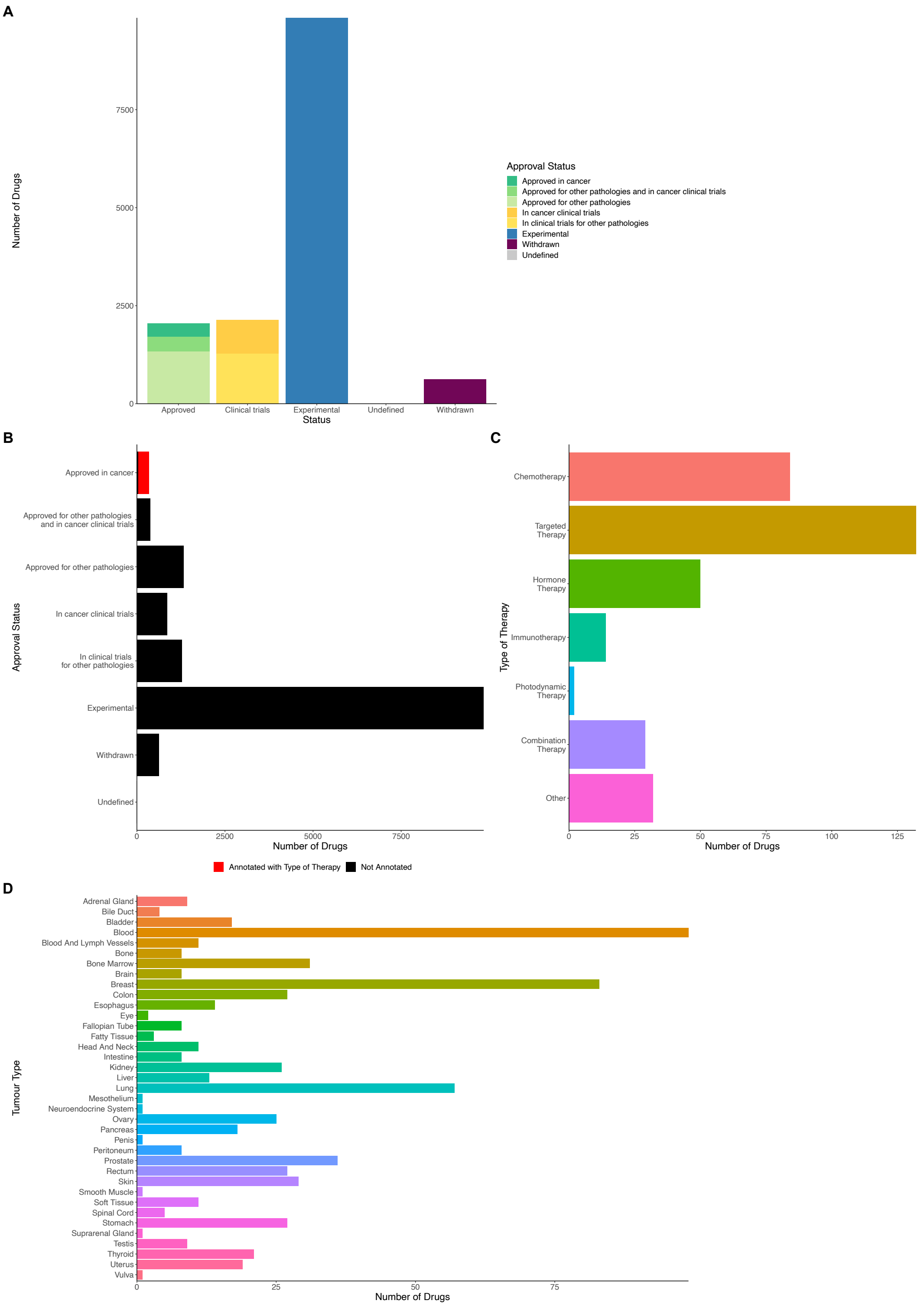

Supplementary Figure S2. PanDrugsdb drug annotations. (A) Number of drugs by approval status. (B) Only drugs approved in cancer have been manually annotated with the type of therapy. (C) Number of drugs approved in cancer by therapy type. (D) Number of drugs approved in cancer by tumour indication.

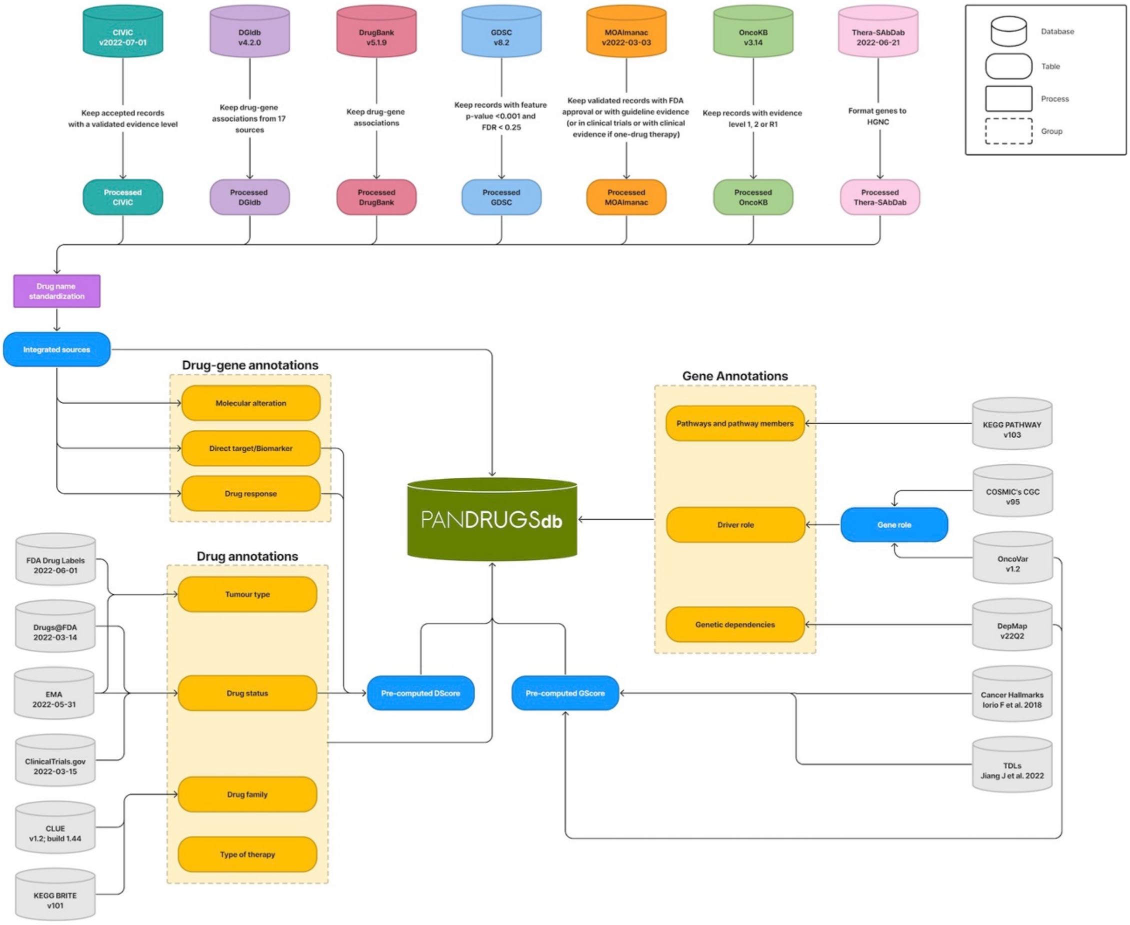

Supplementary Figure S3. PanDrugsdb creation schema. Drug-gene associations were retrieved from 23 sources contained in 7 different source providers. 17 sources were obtained just from DGIdb. After preprocessing each source provider independently, drug names were standardized and all drug-gene associations were integrated into PanDrugsdb. Drug-gene annotations derived from these sources, together with drug annotations obtained from other databases, were used to pre-compute the DScore. Likewise, other databases were mined to obtain gene annotations and pre-compute the GScore. All these annotations, the pre-computed DScore and the pre-computed GScore were also included in PanDrugsdb. The version number, access date or reference of each database is specified below its name. ***DScore:*** *Drug Score;* ***GScore:*** *Gene Score;* ***PanDrugsdb:*** *PanDrugs database. Dates are displayed in ISO 8601 standard format: YYYY-MM-DD.*

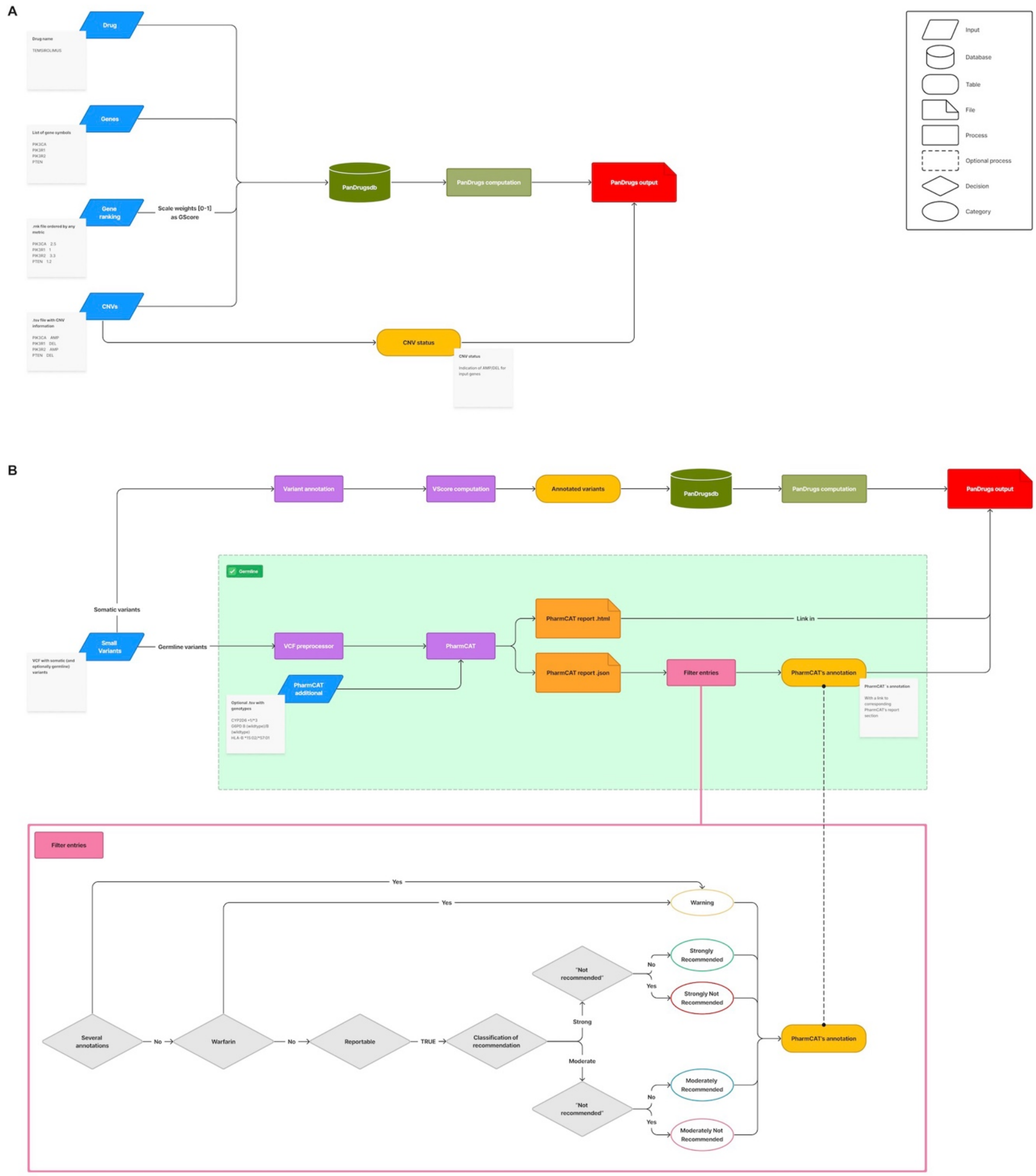

Supplementary Figure S4. PanDrugs2 single-input queries. (A) **In a drug, gene names, gene ranking or CNVs query,** the input list of genes or the input drug is directly used to query PanDrugsdb. After the query, the final DScore and GScores are calculated from the pre-computed scores stored in the database. In a gene ranking query, the final GScore is calculated by scaling the ranking metric between 0 and 1. PanDrugs2 returns a table of prioritized drugs that can contain an additional column detailing the CNV status of each input gene if the user makes a CNVs query. (B) **In a small variants query,** the somatic variants are annotated in order to compute their VScores. Then, the somatic variants with a moderate or high impact are used to query PanDrugsdb. After the query, the final DScore is calculated from the pre-computed one. The GScore is assigned to the maximum VScore among all variants affecting the gene's principal isoform. PanDrugs2 returns a table of prioritized drugs and actionable gene alterations. The user can include germline variants in the input VCF to add pharmacogenetics annotations to PanDrugs2 output. The germline variants are processed before

calling PharmCAT, which returns a report with CPIC guidelines associated with each germline variant. This report is parsed to include a column indicating the relevance of CPIC's recommendation with a link to PharmCAT's report in PanDrugs2 output table. ***AMP:*** *Amplification;* ***CNV:*** *copy number variant;* ***DEL:*** *Deletion;* ***GScore:*** *Gene Score;* ***.html:*** *HyperText Markup Language file;* ***.json:*** *JavaScript Object Notation file;* ***PanDrugsdb:*** *PanDrugs database;* ***PharmCAT:*** *Pharmacogenomics Clinical Annotation Tool;* ***.rnk:*** *Ranked list file;* ***.tsv:*** *Tab Separated Values file;* ***VCF:*** *Variant Calling File;* ***VScore:*** *Variant Score.*

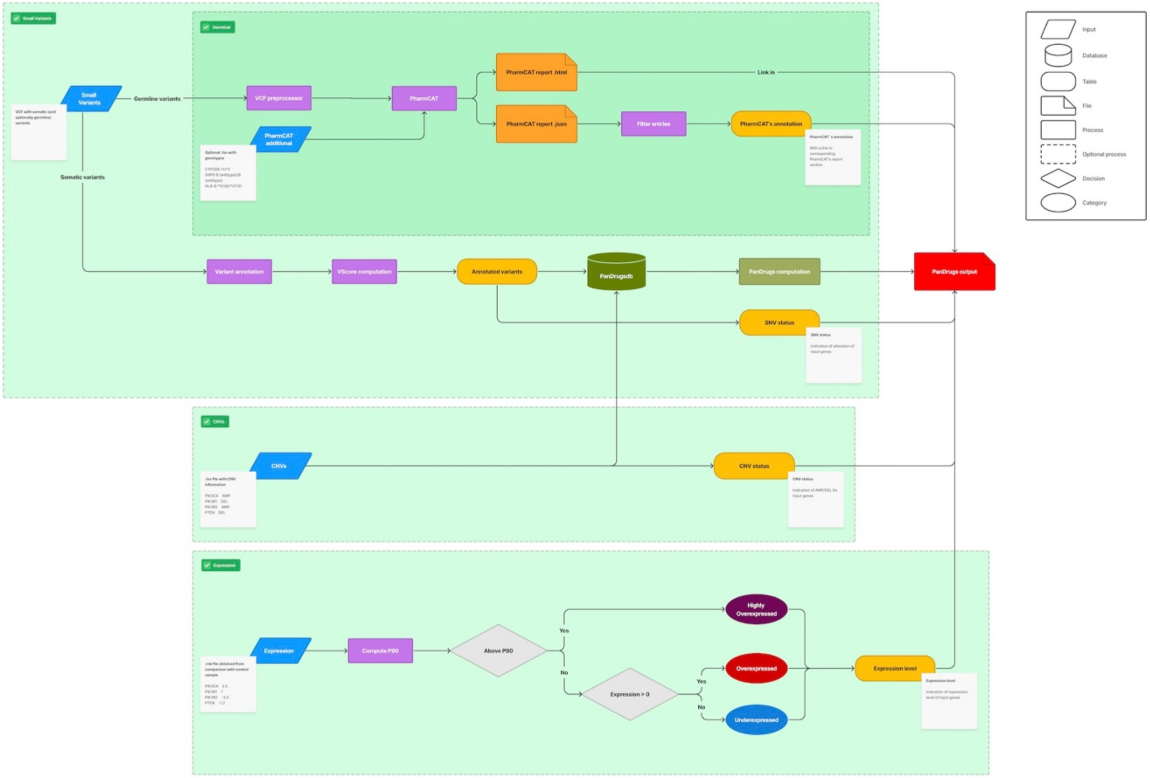

Supplementary Figure S5. PanDrugs2 multi-omics query. In a multi-omics query, the user can use up to 3 different types of omics data: single nucleotide variants, CNVs and an expression ranking obtained from comparison with a control sample. In order to perform a multi-omics query, at least 2 inputs must be supplied. If the input includes single nucleotide variants, the somatic (and optionally germline) variants are processed as indicated in Supplementary Figure S4B. Likewise, if CNV data is available, PanDrugs2 queries PanDrugsdb as explained in Supplementary Figure S4A. Please note that only somatic variants and CNVs are used to query PanDrugsdb. If a gene has small variants and CNVs, the GScore computed from VScores has priority over the other GScore. If expression data is provided, the genes are categorized into 3 levels: highly overexpressed (expression > P90), overexpressed (expression > 0) and underexpressed (expression < 0). PanDrugs2 returns a table of prioritized drugs with up to 4 columns depending on the supplied inputs: a column detailing SNV status of each gene (for somatic variants), another indicating the relevance of CPIC's recommendation with a link to PharmCAT's report (for germline variants), a column with the CNV status of each input gene (for CNV data) and another one indicating the expression level of the queried genes (for expression data). ***AMP:*** *Amplification;* ***CNV:*** *copy number variant;* ***DEL:*** *Deletion;* ***.html:*** *HyperText Markup Language file;* ***.json:*** *JavaScript Object Notation file;* ***PanDrugsdb:*** *PanDrugs database;* ***PharmCAT:*** *Pharmacogenomics Clinical Annotation Tool;* ***P90:*** *90th percentile;* ***.rnk:*** *Ranked list file;* ***SNV:*** *single nucleotide variant;* ***.tsv:*** *Tab Separated Values file;* ***VCF:*** *Variant Calling File;* ***VScore:*** *Variant Score.*

SUPPLEMENTARY TABLES

Source	Source Provider	Version/Access Date	Initial Records	Processed Records	Direct Target/Biomarker	Drug Response	Molecular Alteration	Expertly Curated
CIViC	CIViC	v2022-07-01	4510	32	No	Yes	Yes	Yes
Cancer Commons	DGIdb	v4.2.0	94	93	Yes	No	No	Yes
ChemblInteractions	DGIdb	v4.2.0	7626	7464	Yes	No	No	Yes
Clearity Foundation Biomarkers	DGIdb	v4.2.0	129	129	NA†	NA†	NA†	Yes
Clearity Foundation Clinical Trial	DGIdb	v4.2.0	154	150	NA†	NA†	NA†	Yes
DTC	DGIdb	v4.2.0	24327	23816	Yes	No	Yes	Yes
DoCM	DGIdb	v4.2.0	72	71	No	Yes	Yes	Yes
FDA Pharmacogenomic Biomarkers	DGIdb	v4.2.0	367	367	Yes	Yes	Yes	Yes
Guide to Pharmacology	DGIdb	v4.2.0	5495	5444	Yes	No	No	Yes
JAX-CKB	DGIdb	v4.2.0	1361	1316	No	Yes	Yes	Yes
My Cancer Genome	DGIdb	v4.2.0	804	778	Yes	Yes	Yes	Yes
My Cancer Genome Clinical Trial	DGIdb	v4.2.0	244	229	No	Yes	Yes	Yes
NCI Cancer Gene Index	DGIdb	v4.2.0	3416	3349	Yes	Yes	Yes	Yes
PharmGKB	DGIdb	v4.2.0	4195	4135	No	No	Yes	No

TALC	DGIdb	v4.2.0	440	431	Yes	Yes	No	Yes
TEND	DGIdb	v4.2.0	2143	2116	Yes	No	No	Yes
TTD	DGIdb	v4.2.0	3745	3648	Yes	Yes	Yes	No
TdgClinicalTrial	DGIdb	v4.2.0	3903	3809	Yes	No	No	Yes
DrugBank	DrugBank	v5.1.9	14303	14274	Yes	Yes	Yes	Yes
GDSC	GDSC	v8.2	1041*	1025	No	Yes	Yes	No
MOAlmanac	MOAlmanac	v2022-03-03	820	149	No	Yes	Yes	Yes
OncoKB	OncoKB	v3.14	654	121	No	Yes	Yes	Yes
Thera-SAbDab	Thera-SAbDab	2022-06-21	748	765	Yes	No	No	No

Supplementary Table S1. PanDrugsdb drug-gene association sources. The table shows each source's provider, version number or access date, the number of initial records, the number of processed records stored in PanDrugsdb, whether the source contains annotations of direct targets or biomarkers, drug response (sensitivity or resistance) or molecular alterations associated with drug response and whether they are expertly curated or not. ***:** *After p-value and FDR filtering.* ***NA†:*** *As DGIdb states, data is no longer publicly available from the source. Dates are displayed in ISO 8601 standard format: YYYY-MM-DD.*

Disease	Drug Status	Druggable Gene	Pre-computed DScore
Cancer	Approved	Direct target	±1
		Biomarker	±0.9
Other (in cancer clinical trials)		Direct target	±0.8
		Biomarker	±0.7
Cancer	Clinical Trials	Direct target	±0.6
		Biomarker	±0.5
Other	Approved	Direct target	±0.4
		Biomarker	±0.3
	Clinical Trials	Direct target	±0.2
		Biomarker	±0.1
	Experimental	Direct target	±0.0008
		Biomarker	±0.0004

Supplementary Table S2. Features contributing to pre-computed DScore calculation.

Feature	Value	Score	Weight	Score component
**DepMap Gene Effect**	Skewness > -0.5	0	0.15	Cancer Essentiality and Tumor Vulnerability
	-0.5 ≥ Skewness ≥ -2	Skewness [0-1]		
	Skewness < -2	1		
**OncoVar**	Driver Level ≤ 3	Consensus Score [0-0.5]	0.55	Relevance in Cancer
	Driver Level = 4	Consensus Score [0.5-1]		
**Cancer Hallmarks**	# Hallmarks > 5	1	0.1	
	# Hallmarks ≤ 5	0.2 · # Hallmarks		
**TDLs**	Tclin	1	0.2	Druggability
	Tchem	0.5		
	Tbio	0.25		
	Tdark	0.125		

Supplementary Table S3. Features contributing to pre-computed GScore calculation. The table shows the assigned score for each feature value, the weight of each feature and the GScore component to which each feature contributes. ***#:** Number.*

Feature	Value	ONC Weighted Score	TSG Weighted Score	Score Component
**Pre-computed GScore**	[0-1]	+0.125 · GScore	+0.125 · GScore	Cancer Essentiality and Tumor Vulnerability; Relevance in Cancer; Druggability
**PolyPhen score**	> 0.435	n: # of predictors that meet the criteria<br>If n ≥ 3: +0.125<br>If n = 2: +0.080<br>If n = 1: +0.040	n: # of predictors that meet the criteria<br>If n ≥ 3: +0.125<br>If n = 2: +0.080<br>If n = 1: +0.040	Biological impact
**SIFT score**	≤ 0.05			
**CADD score**	> 20			
**FATHMM prediction**	Pathogenic			
**VEP Variant Impact**	High	+0.125	+0.125	
**Domains**	Listed as relevant in cancer* or is a stop-gain/frameshift variant found before the last protein domain	+0.125	+0.125	
	Found within a domain in other circumstances	+0.125/2	+0.125/2	
**Zygosity**	Homozygous	+0.125	+0.1875	
**GMAF**	< 1	+0.125/2	+0.125/2	Frequency
**gnomAD**	< 1	+0.125/2	+0.125/2	
**Mutation frequency in COSMIC**	≥ 100	+0.125/2		
	< 100	+(0.125/2) · (log(freq) / log(max(freq)))		
**Gene frequency in COSMIC**	≥ 100	+0.125/2	+0.125/2	
	< 100	+(0.125/2) · (log(freq) / log(max(freq)))	+(0.125/2) · (log(freq) / log(max(freq)))	
**ClinVar**	Pathogenic with zygosity data	+0.125	+0.125	Clinical Implication
	Pathogenic without zygosity data	+0.250	+0.3125	

Supplementary Table S4. Annotations contributing to VScore calculation. The table shows the assigned weighted score for each feature value depending on the driver role of the gene, together with the VScore component to which each feature contributes.

SUPPLEMENTARY REFERENCES

1. Griffith,M., Spies,N.C., Krysiak,K., McMichael,J.F., Coffman,A.C., Danos,A.M., Ainscough,B.J., Ramirez,C.A., Rieke,D.T., Kujan,L., *et al.* (2017) CIViC is a community knowledgebase for expert crowdsourcing the clinical interpretation of variants in cancer. *Nat. Genet.*, **49**, 170–174. PMID: 28138153. DOI: 10.1038/ng.3774. PMCID: PMC5367263.

2. Freshour,S.L., Kiwala,S., Cotto,K.C., Coffman,A.C., McMichael,J.F., Song,J.J., Griffith,M., Griffith,O.L. and Wagner,A.H. (2021) Integration of the Drug-Gene Interaction Database (DGIdb 4.0) with open crowdsource efforts. *Nucleic Acids Res.*, **49**, D1144–D1151. PMID: 33237278. DOI: 10.1093/nar/gkaa1084. PMCID: PMC7778926.

3. Frankish,A., Diekhans,M., Jungreis,I., Lagarde,J., Loveland,J.E., Mudge,J.M., Sisu,C., Wright,J.C., Armstrong,J., Barnes,I., *et al.* (2021) GENCODE 2021. *Nucleic Acids Res.*, **49**, D916–D923. PMID: 33270111. DOI: 10.1093/nar/gkaa1087. PMCID: PMC7778937.

4. Shrager,J., Tenenbaum,J.M. and Travers,M. (2011) Cancer Commons: Biomedicine in the Internet Age. In *Collaborative Computational Technologies for Biomedical Research*.pp. 161–177. DOI: 10.1002/9781118026038.ch11.

5. Mendez,D., Gaulton,A., Bento,A.P., Chambers,J., De Veij,M., Félix,E., Magariños,M.P., Mosquera,J.F., Mutowo,P., Nowotka,M., *et al.* (2019) ChEMBL: towards direct deposition of bioassay data. *Nucleic Acids Res.*, **47**, D930–D940. PMID: 30398643. DOI: 10.1093/nar/gky1075. PMCID: PMC6323927.

6. Clearity Foundation (2013) Clearity Foundation Biomarkers.

7. Clearity Foundation (2013) Clearity Foundation Clinical Trial.

8. Ainscough,B.J., Griffith,M., Coffman,A.C., Wagner,A.H., Kunisaki,J., Choudhary,M.N., McMichael,J.F., Fulton,R.S., Wilson,R.K., Griffith,O.L., *et al.* (2016) DoCM: a database of curated mutations in cancer. *Nat. Methods*, **13**, 806–807. PMID: 27684579. DOI: 10.1038/nmeth.4000. PMCID: PMC5317181.

9. Tanoli,Z., Alam,Z., Vähä-Koskela,M., Ravikumar,B., Malyutina,A., Jaiswal,A., Tang,J., Wennerberg,K. and Aittokallio,T. (2018) Drug Target Commons 2.0: a community platform for systematic analysis of drug-target interaction profiles. *Database* , **2018**, 1–13. PMID: 30219839. DOI: 10.1093/database/bay083. PMCID: PMC6146131.

10. U.S. Food and Drug Administration (2020) Table of Pharmacogenomic Biomarkers in Drug Labeling.

11. Armstrong,J.F., Faccenda,E., Harding,S.D., Pawson,A.J., Southan,C., Sharman,J.L., Campo,B., Cavanagh,D.R., Alexander,S.P.H., Davenport,A.P., *et al.* (2020) The IUPHAR/BPS Guide to PHARMACOLOGY in 2020: extending immunopharmacology content and introducing the IUPHAR/MMV Guide to MALARIA PHARMACOLOGY. *Nucleic Acids Res.*, **48**, D1006–D1021. PMID: 31691834. DOI: 10.1093/nar/gkz951. PMCID: PMC7145572.

12. Patterson,S.E., Liu,R., Statz,C.M., Durkin,D., Lakshminarayana,A. and Mockus,S.M. (2016) The clinical trial landscape in oncology and connectivity of somatic mutational profiles to targeted therapies. *Hum. Genomics*, **10**, 4. PMID: 26772741. DOI: 10.1186/s40246-016-0061-7. PMCID: PMC4715272.

13. Jain,N., Mittendorf,K.F., Holt,M., Lenoue-Newton,M., Maurer,I., Miller,C., Stachowiak,M., Botyrius,M., Cole,J., Micheel,C., *et al.* (2020) The My Cancer Genome clinical trial data model and trial curation workflow. *J. Am. Med. Inform. Assoc.*, **27**, 1057–1066. PMID: 32483629. DOI: 10.1093/jamia/ocaa066. PMCID: PMC7647323.

14. National Cancer Institute (2017) NCI Cancer Gene Index.

15. Whirl-Carrillo,M., Huddart,R., Gong,L., Sangkuhl,K., Thorn,C.F., Whaley,R. and Klein,T.E. (2021) An Evidence-Based Framework for Evaluating Pharmacogenomics Knowledge for Personalized Medicine. *Clin. Pharmacol. Ther.*, **110**, 563–572. PMID: 34216021. DOI: 10.1002/cpt.2350. PMCID: PMC8457105.

16. Morgensztern,D., Campo,M.J., Dahlberg,S.E., Doebele,R.C., Garon,E., Gerber,D.E., Goldberg,S.B., Hammerman,P.S., Heist,R.S., Hensing,T., *et al.* (2015) Molecularly targeted therapies in non-small-cell lung cancer annual update 2014. *J. Thorac. Oncol.*, **10**, S1–63. PMID: 25535693. DOI: 10.1097/JTO.0000000000000405. PMCID: PMC4346098.

17. Rask-Andersen,M., Masuram,S. and Schiöth,H.B. (2014) The druggable genome: Evaluation of drug targets in clinical trials suggests major shifts in molecular class and indication. *Annu. Rev. Pharmacol. Toxicol.*, **54**, 9–26. PMID: 24016212. DOI: 10.1146/annurev-pharmtox-011613-135943.

18. Rask-Andersen,M., Almén,M.S. and Schiöth,H.B. (2011) Trends in the exploitation of novel drug targets. *Nat. Rev. Drug Discov.*, **10**, 579–590. PMID: 21804595. DOI: 10.1038/nrd3478.

19. Wang,Y., Zhang,S., Li,F., Zhou,Y., Zhang,Y., Wang,Z., Zhang,R., Zhu,J., Ren,Y., Tan,Y., *et al.* (2020) Therapeutic target database 2020: enriched resource for facilitating research and early development of targeted therapeutics. *Nucleic Acids Res.*, **48**, D1031–D1041. PMID: 31691823. DOI: 10.1093/nar/gkz981. PMCID: PMC7145558.

20. Wishart,D.S., Feunang,Y.D., Guo,A.C., Lo,E.J., Marcu,A., Grant,J.R., Sajed,T., Johnson,D., Li,C., Sayeeda,Z., *et al.* (2018) DrugBank 5.0: a major update to the DrugBank database for 2018. *Nucleic Acids Res.*, **46**, D1074–D1082. PMID: 29126136. DOI: 10.1093/nar/gkx1037. PMCID: PMC5753335.

21. DrugBank Database XML Parser *Paperpile*.

22. Iorio,F., Knijnenburg,T.A., Vis,D.J., Bignell,G.R., Menden,M.P., Schubert,M., Aben,N., Gonçalves,E., Barthorpe,S., Lightfoot,H., *et al.* (2016) A Landscape of Pharmacogenomic Interactions in Cancer. *Cell*, **166**, 740–754. DOI: 10.1016/j.cell.2016.06.017.

23. Reardon,B., Moore,N.D., Moore,N.S., Kofman,E., AlDubayan,S.H., Cheung,A.T.M., Conway,J., Elmarakeby,H., Imamovic,A., Kamran,S.C., *et al.* (2021) Integrating molecular profiles into clinical frameworks through the Molecular Oncology Almanac to prospectively guide precision oncology. *Nat Cancer*, **2**, 1102–1112. PMID: 35121878. DOI: 10.1038/s43018-021-00243-3. PMCID: PMC9082009.

24. Chakravarty,D., Gao,J., Phillips,S.M., Kundra,R., Zhang,H., Wang,J., Rudolph,J.E., Yaeger,R., Soumerai,T., Nissan,M.H., *et al.* (2017) OncoKB: A Precision Oncology Knowledge Base. *JCO Precis Oncol*, **2017**. PMID: 28890946. DOI: 10.1200/PO.17.00011. PMCID: PMC5586540.

25. Raybould,M.I.J., Marks,C., Lewis,A.P., Shi,J., Bujotzek,A., Taddese,B. and Deane,C.M. (2020) Thera-SAbDab: the Therapeutic Structural Antibody Database. *Nucleic Acids Res.*, **48**, D383–D388. PMID: 31555805. DOI: 10.1093/nar/gkz827. PMCID: PMC6943036.

26. Seal,R.L., Braschi,B., Gray,K., Jones,T.E.M., Tweedie,S., Haim-Vilmovsky,L. and Bruford,E.A. (2023) Genenames.org: the HGNC resources in 2023. *Nucleic Acids Res.*, **51**, D1003–D1009. PMID: 36243972. DOI: 10.1093/nar/gkac888. PMCID: PMC9825485.

27. Swain,M. (2017) PubChemPy.

28. Kim,S., Chen,J., Cheng,T., Gindulyte,A., He,J., He,S., Li,Q., Shoemaker,B.A., Thiessen,P.A., Yu,B., *et al.* (2023) PubChem 2023 update. *Nucleic Acids Res.*, **51**, D1373–D1380. PMID: 36305812. DOI: 10.1093/nar/gkac956. PMCID: PMC9825602.

29. U.S. Food and Drug Administration (2022) Drugs@FDA.

30. European Medicines Agency European Public Assessment Reports.

31. ClinicalTials.gov ClinicalTrials.gov Database.

32. U.S. Food and Drug Administration Drug Labeling.

33. Kanehisa,M., Furumichi,M., Sato,Y., Kawashima,M. and Ishiguro-Watanabe,M. (2023) KEGG for taxonomy-based analysis of pathways and genomes. *Nucleic Acids Res.*, **51**, D587–D592. PMID: 36300620. DOI: 10.1093/nar/gkac963. PMCID: PMC9825424.

34. Subramanian,A., Narayan,R., Corsello,S.M., Peck,D.D., Natoli,T.E., Lu,X., Gould,J., Davis,J.F., Tubelli,A.A., Asiedu,J.K., *et al.* (2017) A Next Generation Connectivity Map: L1000 Platform and the First 1,000,000 Profiles. *Cell*, **171**, 1437–1452.e17. PMID: 29195078. DOI: 10.1016/j.cell.2017.10.049. PMCID: PMC5990023.

35. Sondka,Z., Bamford,S., Cole,C.G., Ward,S.A., Dunham,I. and Forbes,S.A. (2018) The COSMIC Cancer Gene Census: describing genetic dysfunction across all human cancers. *Nat. Rev. Cancer*, **18**, 696–705. PMID: 30293088. DOI: 10.1038/s41568-018-0060-1. PMCID: PMC6450507.

36. Wang,T., Ruan,S., Zhao,X., Shi,X., Teng,H., Zhong,J., You,M., Xia,K., Sun,Z. and Mao,F. (2021) OncoVar: an integrated database and analysis platform for oncogenic driver variants in cancers. *Nucleic Acids Res.*, **49**, D1289–D1301. PMID: 33179738. DOI: 10.1093/nar/gkaa1033. PMCID: PMC7778899.

37. Cancer Genome Atlas Research Network, Weinstein,J.N., Collisson,E.A., Mills,G.B., Shaw,K.R.M., Ozenberger,B.A., Ellrott,K., Shmulevich,I., Sander,C. and Stuart,J.M. (2013) The Cancer Genome Atlas Pan-Cancer analysis project. *Nat. Genet.*, **45**, 1113–1120. PMID: 24071849. DOI: 10.1038/ng.2764. PMCID: PMC3919969.

38. Vogelstein,B., Papadopoulos,N., Velculescu,V.E., Zhou,S., Diaz,L.A.,Jr and Kinzler,K.W. (2013) Cancer genome landscapes. *Science*, **339**, 1546–1558. PMID: 23539594. DOI: 10.1126/science.1235122. PMCID: PMC3749880.

39. Bailey,M.H., Tokheim,C., Porta-Pardo,E., Sengupta,S., Bertrand,D., Weerasinghe,A., Colaprico,A., Wendl,M.C., Kim,J., Reardon,B., *et al.* (2018) Comprehensive Characterization of Cancer Driver Genes and Mutations. *Cell*, **173**, 371–385.e18. PMID: 29625053. DOI: 10.1016/j.cell.2018.02.060. PMCID: PMC6029450.

40. Liu,Y., Sun,J. and Zhao,M. (2017) ONGene: A literature-based database for human oncogenes. *J. Genet. Genomics*, **44**, 119–121. PMID: 28162959. DOI: 10.1016/j.jgg.2016.12.004.

41. Zhao,M., Kim,P., Mitra,R., Zhao,J. and Zhao,Z. (2016) TSGene 2.0: an updated literature-based knowledgebase for tumor suppressor genes. *Nucleic Acids Res.*, **44**, D1023–31. PMID: 26590405. DOI: 10.1093/nar/gkv1268. PMCID: PMC4702895.

42. Rian,K., Hidalgo,M.R., Çubuk,C., Falco,M.M., Loucera,C., Esteban-Medina,M., Alamo-Alvarez,I., Peña-Chilet,M. and Dopazo,J. (2021) Genome-scale mechanistic modeling of signaling pathways made easy: A bioconductor/cytoscape/web server framework for the analysis of omic data. *Comput. Struct. Biotechnol. J.*, **19**, 2968–2978. PMID: 34136096. DOI: 10.1016/j.csbj.2021.05.022. PMCID: PMC8170118.

43. Perales-Patón,J., Di Domenico,T., Fustero-Torre,C., Piñeiro-Yáñez,E., Carretero-Puche,C., Tejero,H., Valencia,A., Gómez-López,G. and Al-Shahrour,F. (2019) vulcanSpot: a tool to prioritize therapeutic vulnerabilities in cancer. *Bioinformatics*, **35**, 4846–4848. PMID: 31173067. DOI: 10.1093/bioinformatics/btz465. PMCID: PMC6853644.

44. DepMap,B. (2022) DepMap 22Q2 Public.

45. Zhao,H., Sun,Z., Wang,J., Huang,H., Kocher,J.-P. and Wang,L. (2014) CrossMap: a versatile tool for coordinate conversion between genome assemblies. *Bioinformatics*, **30**, 1006–1007. PMID: 24351709. DOI: 10.1093/bioinformatics/btt730. PMCID: PMC3967108.

46. Korotkevich,G., Sukhov,V., Budin,N., Shpak,B., Artyomov,M.N. and Sergushichev,A. (2021) Fast gene set enrichment analysis. *bioRxiv*, 10.1101/060012. DOI: 10.1101/060012.

47. Dempster,J.M., Boyle,I., Vazquez,F., Root,D.E., Boehm,J.S., Hahn,W.C., Tsherniak,A. and McFarland,J.M. (2021) Chronos: a cell population dynamics model of CRISPR experiments that improves inference of gene fitness effects. *Genome Biol.*, **22**, 343. PMID: 34930405. DOI: 10.1186/s13059-021-02540-7. PMCID: PMC8686573.

48. Iorio,F., Garcia-Alonso,L., Brammeld,J.S., Martincorena,I., Wille,D.R., McDermott,U. and Saez-Rodriguez,J. (2018) Pathway-based dissection of the genomic heterogeneity of cancer hallmarks' acquisition with SLAPenrich. *Sci. Rep.*, **8**, 6713. PMID: 29713020. DOI: 10.1038/s41598-018-25076-6. PMCID: PMC5928049.

49. Jiang,J., Yuan,J., Hu,Z., Zhang,Y., Zhang,T., Xu,M., Long,M., Fan,Y., Tanyi,J.L., Montone,K.T., *et al.* (2022) Systematic illumination of druggable genes in cancer genomes. *Cell Rep.*, **38**, 110400. PMID: 35196490. DOI: 10.1016/j.celrep.2022.110400. PMCID: PMC8919705.

50. McLaren,W., Gil,L., Hunt,S.E., Riat,H.S., Ritchie,G.R.S., Thormann,A., Flicek,P. and Cunningham,F. (2016) The Ensembl Variant Effect Predictor. *Genome Biol.*, **17**, 122. PMID: 27268795. DOI: 10.1186/s13059-016-0974-4. PMCID: PMC4893825.

51. Yang,F., Petsalaki,E., Rolland,T., Hill,D.E., Vidal,M. and Roth,F.P. (2015) Protein domain-level landscape of cancer-type-specific somatic mutations. *PLoS Comput. Biol.*, **11**, e1004147. PMID: 25794154. DOI: 10.1371/journal.pcbi.1004147. PMCID: PMC4368709.

52. Landrum,M.J., Lee,J.M., Benson,M., Brown,G.R., Chao,C., Chitipiralla,S., Gu,B., Hart,J., Hoffman,D., Jang,W., *et al.* (2018) ClinVar: improving access to variant interpretations and supporting evidence. *Nucleic Acids Res.*, **46**, D1062–D1067. PMID: 29165669. DOI: 10.1093/nar/gkx1153. PMCID: PMC5753237.

53. Tate,J.G., Bamford,S., Jubb,H.C., Sondka,Z., Beare,D.M., Bindal,N., Boutselakis,H., Cole,C.G., Creatore,C., Dawson,E., *et al.* (2019) COSMIC: the Catalogue Of Somatic Mutations In Cancer. *Nucleic Acids Res.*, **47**, D941–D947. PMID: 30371878. DOI: 10.1093/nar/gky1015. PMCID: PMC6323903.

54. Paysan-Lafosse,T., Blum,M., Chuguransky,S., Grego,T., Pinto,B.L., Salazar,G.A., Bileschi,M.L., Bork,P., Bridge,A., Colwell,L., *et al.* (2023) InterPro in 2022. *Nucleic Acids Res.*, **51**, D418–D427. PMID: 36350672. DOI: 10.1093/nar/gkac993. PMCID: PMC9825450.

55. Mistry,J., Chuguransky,S., Williams,L., Qureshi,M., Salazar,G.A., Sonnhammer,E.L.L., Tosatto,S.C.E., Paladin,L., Raj,S., Richardson,L.J., *et al.* (2021) Pfam: The protein families database in 2021. *Nucleic Acids Res.*, **49**, D412–D419. PMID: 33125078. DOI: 10.1093/nar/gkaa913. PMCID: PMC7779014.

56. UniProt Consortium (2023) UniProt: the Universal Protein Knowledgebase in 2023. *Nucleic Acids Res.*, **51**, D523–D531. PMID: 36408920. DOI: 10.1093/nar/gkac1052. PMCID: PMC9825514.

57. Rodriguez,J.M., Pozo,F., Cerdán-Vélez,D., Di Domenico,T., Vázquez,J. and Tress,M.L. (2022) APPRIS: selecting functionally important isoforms. *Nucleic Acids Res.*, **50**, D54–D59. PMID: 34755885. DOI: 10.1093/nar/gkab1058. PMCID: PMC8728124.

58. Sangkuhl,K., Whirl-Carrillo,M., Whaley,R.M., Woon,M., Lavertu,A., Altman,R.B., Carter,L., Verma,A., Ritchie,M.D. and Klein,T.E. (2020) Pharmacogenomics Clinical Annotation Tool (PharmCAT). *Clin. Pharmacol. Ther.*, **107**, 203–210. PMID: 31306493. DOI: 10.1002/cpt.1568. PMCID: PMC6977333.

59. Relling,M.V. and Klein,T.E. (2011) CPIC: Clinical Pharmacogenetics Implementation Consortium of the Pharmacogenomics Research Network. *Clin. Pharmacol. Ther.*, **89**, 464–467. PMID: 21270786. DOI: 10.1038/clpt.2010.279. PMCID: PMC3098762.

60. Grossman,R.L., Heath,A.P., Ferretti,V., Varmus,H.E., Lowy,D.R., Kibbe,W.A. and Staudt,L.M. (2016) Toward a Shared Vision for Cancer Genomic Data. *N. Engl. J. Med.*, **375**, 1109–1112. PMID: 27653561. DOI: 10.1056/NEJMp1607591. PMCID: PMC6309165.

61. Piñeiro-Yáñez,E., Reboiro-Jato,M., Gómez-López,G., Perales-Patón,J., Troulé,K., Rodríguez,J.M., Tejero,H., Shimamura,T., López-Casas,P.P., Carretero,J., *et al.* (2018) PanDrugs: a novel method to prioritize anticancer drug treatments according to individual genomic data. *Genome Med.*, **10**, 41. PMID: 29848362. DOI: 10.1186/s13073-018-0546-1. PMCID: PMC5977747.

62. Cerami,E., Gao,J., Dogrusoz,U., Gross,B.E., Sumer,S.O., Aksoy,B.A., Jacobsen,A., Byrne,C.J., Heuer,M.L., Larsson,E., *et al.* (2012) The cBio cancer genomics portal: an open platform for exploring multidimensional cancer genomics data. *Cancer Discov.*, **2**, 401–404. PMID: 22588877. DOI: 10.1158/2159-8290.CD-12-0095. PMCID: PMC3956037.

63. Huang,K.-L., Mashl,R.J., Wu,Y., Ritter,D.I., Wang,J., Oh,C., Paczkowska,M., Reynolds,S., Wyczalkowski,M.A., Oak,N., *et al.* (2018) Pathogenic Germline Variants in 10,389 Adult Cancers. *Cell*, **173**, 355–370.e14. PMID: 29625052. DOI: 10.1016/j.cell.2018.03.039. PMCID: PMC5949147.
